# Effects of potential risk factors on the development of cardiometabolic multimorbidity and mortality among the elders in China

**DOI:** 10.3389/fcvm.2022.966217

**Published:** 2022-09-09

**Authors:** Huihui Zhang, Xinyu Duan, Peixi Rong, Yusong Dang, Mingxin Yan, Yaling Zhao, Fangyao Chen, Jing Zhou, Yulong Chen, Duolao Wang, Leilei Pei

**Affiliations:** ^1^Department of Epidemiology and Health Statistics, School of Public Health, Xi’an Jiaotong University Health Science Center, Xi’an, China; ^2^Department of Pediatrics, The Second Affiliated Hospital of Xi’an Jiaotong University, Xi’an, China; ^3^Shaanxi Key Laboratory of Ischemic Cardiovascular Disease, Shaanxi Key Laboratory of Brain Disorders, Institute of Basic and Translational Medicine, Xi’an Medical University, Xi’an, China; ^4^Biostatistics Unit, Department of Clinical Sciences, Liverpool School of Tropical Medicine, Liverpool, United Kingdom

**Keywords:** multi-state Markov model, cardiometabolic disease, multimorbidity, economic status, behavior lifestyle

## Abstract

**Objectives:**

To examine the impact of demographic, socioeconomic, and behavioral factors on the development of cardiometabolic multimorbidity and mortality in Chinese elders.

**Methods:**

Data from the Chinese Longitudinal Healthy Longevity Survey (CLHLS) 2002–2018 was used in the study. Cardiometabolic multimorbidity was defined as the presence of two or more cardiometabolic disorders, such as hypertension, diabetes, cardiovascular disease (CVD), heart disease, or stroke. Cox regression model and multi-state Markov model were developed to evaluate the association of the study factors with the progression of cardiometabolic conditions and mortality. The outcomes included three states (first cardiometabolic disease, cardiometabolic multimorbidity, and all-cause mortality) and five possible transitions among the three states.

**Results:**

Of the 13,933 eligible individuals, 7,917 (56.8%) were female, and 9,540 (68.50%) were over 80 years old. 2,766 (19.9%) participants had their first cardiometabolic disease, 975 (7.0%) participants suffered from cardiometabolic multimorbidity, and 9,365 (67.2%) participants died. The progression to cardiometabolic multimorbidity was positively associated with being female (HR = 1.42; 95%CI, 1.10 − 1.85), living in the city (HR = 1.41; 95%CI, 1.04 − 1.93), overweight (HR = 1.43; 95%CI, 1.08 − 1.90), and obesity (HR = 1.75; 95% CI, 1.03 − 2.98). A higher risk for the first cardiometabolic disease was associated with being female (HR = 1.26; 95% CI, 1.15 − 1.39), higher socioeconomic status (SES, HR = 1.17; 95%CI, 1.07 − 1.28), lack of regular physical activity (HR = 1.13; 95%CI, 1.04 − 1.23), smoking (HR = 1.20; 95%CI, 1.08 − 1.33), ≤ 5 h sleep time (HR = 1.15; 95%CI, 1.02 − 1.30), overweight (HR = 1.48; 95% CI, 1.32 − 1.66), and obesity (HR = 1.34; 95%CI, 1.06 − 1.69). It also should be noted that not in marriage, lower SES and unhealthy behavioral patterns were risk factors for mortality.

**Conclusion:**

This study emphasized the importance of lifestyle and SES in tackling the development of cardiometabolic conditions among Chinese elders and provided a reference for policy-makers to develop a tailored stage-specific intervention strategy.

## Introduction

Cardiometabolic multimorbidity is commonly defined as the simultaneous presence of two or more cardiometabolic disorders, such as hypertension, diabetes, cardiovascular disease (CVD), heart disease, or stroke) (1–3). Considerable evidence confirms the negative impact of cardiometabolic multimorbidity on patients, family, and healthcare systems, including shorter life expectancy, worse cognitive function, and great medical burden (4–6). It is estimated that the worldwide prevalence of multimorbidity ranges from 12.9% in the general population to 95.1% in the population 65 years and older (7). With the rapid urbanization, the economic transition, and Western lifestyle popularization, the prevalence of non-communicable multimorbidity is rising in low- and middle-income countries (LMICs). According to the reviews of Abebe et al. the prevalence of multimorbidity in LMICs has increased from 3.2 to 67.8% among people over 18 years old (8). Meta-analyses have shown a 43% of prevalence of multimorbidity of chronic conditions in Latin America and the Caribbean (9, 10). Yao et al. have shown that multimorbidity occurred in 42.4% of the participants aged at least 50 years in China in 2011–2015 (11). Moreover, the burden of cardiometabolic diseases rises rapidly in LMICs and is further aggravated by the fragile health and social protection systems (12). This implies that more sophisticated measurements and management of cardiometabolic multimorbidity may have important implications on individual, clinical, and public health in LMICs.

With the continuous increment of life expectancy (13), population aging has contributed substantially to the development of multimorbidity of chronic conditions. It is estimated that people 65 years or over will reach 727 million worldwide in 2020 and 80% of the elderly population is projected to live in LMICs by 2050 (12). Approximately 260 million people aged 60 and above lived in China in 2020, and the share of the elderly population is expected to increase to one-third by 2050. This phenomenon will inevitably bring about a growing burden of non-communicable multimorbidity in LMICs. In particular, the prevalence of cardiometabolic multimorbidity has been increasing rapidly in recent years (14, 15). As a result, the increase in cardiometabolic multimorbidity among older adults could pose a barrier to the development of LMICs, especially in China, leading to a higher medical burden.

Conventional survival analyses have been widely used in previous studies to explore the relationship between risk factors and cardiometabolic diseases (1, 15–17). However, the progress of cardiometabolic multimorbidity is in succession from a healthy state to the first cardiometabolic disease, cardiometabolic multimorbidity, and death. To date, information on how the risk factors impact this sequential progress from a longitudinal perspective is limited. In 2018, one study explored the progress of cardiometabolic multimorbidity in the United Kingdom, but it only included diabetes, coronary heart disease, and stroke (18). Unfortunately, similar studies have not been found among older adults in LMICs, such as in China. Therefore, clarifying the successive progression of cardiometabolic multimorbidity will provide targeted preventive, diagnostic, prognostic, and treatment strategies for older adults. Therefore, the study aimed to analyze how risk factors impact the course of cardiometabolic multimorbidity among Chinese elders during the 2002–2018 period.

## Materials and methods

### Study design and participants

This study data was from the Chinese Longitudinal Healthy Longevity Survey (CLHLS), an ongoing mixed longitudinal cohort established by Duke University and Peking University in 1998 to investigate health-related factors among Chinese elders.^1^ Participants were randomly selected from about half of the cities/counties in 23 Chinese provinces, with a total of 113,000 household visits (19). Data were collected using the structured questionnaire and clinical evaluation every 2 − 3 years. Details of the study design and data collection have been described previously (20–22). CLHLS procedures were approved by the Research Ethics Committees of Duke University and Peking University (IRB00001052-13074). The informed consent of participants and research ethics approval were renewed at each follow-up.

**FIGURE 1:**
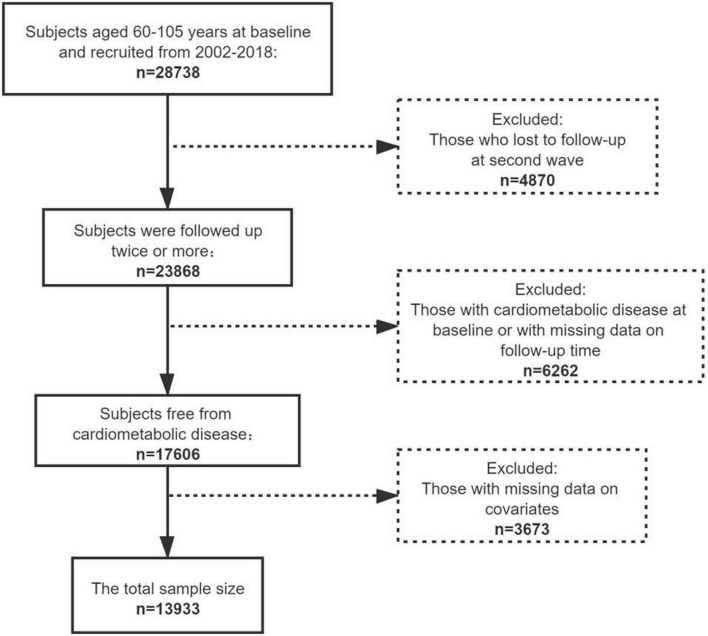
Flowchart of sample selection.

The present study targeted 60–105-years-old participants with no cardiometabolic diseases at the baseline and at least one follow-up record with a definite follow-up time. Due to lacking information on participants’ height and weight between 1998 and 2000, we extracted 28,738 participants from CLHLS 2002–2018. Among these participants, 4,870 were excluded as they were lost to follow-up at second wave, and 6,262 participants were excluded because they had cardiometabolic disease at the baseline or lacked a definite follow-up time. Participants with missing information on smoking (*n* = 13), alcohol consumption (*n* = 21), BMI (*n* = 2,512) and other covariates (*n* = 1,127) were also excluded. Ultimately, 13,933 participants were included in the final study population. Figure 1 shows the participant flowchart.

### Outcomes ascertainment

First cardiometabolic disease, cardiometabolic multimorbidity, and mortality were the main outcome variables in the study. Self-reported information on cardiometabolic conditions was collected by trained research assistants. At each investigation, participants were asked, “Have you ever been told by a doctor that you are suffering from hypertension, diabetes, heart disease, stroke, or CVD?” There were three possible answers for each disease: yes, no, and do not know. The first cardiometabolic disease was identified as having only one of the four diseases mentioned above. Cardiometabolic multimorbidity was identified as having at least two of the four diseases. For participants who died during the study, the details before death were collected in interviews with one of their close family members.

### Socioeconomic status assessment

In this study, six economy-related variables were used to measure socioeconomic status (SES). Education years were re-categorized into four levels: no education (schooling years = 0), elementary school (schooling years = 1 − 5), secondary school (schooling years = 6 − 12), and university or above (schooling years ≥ 13). Participants were asked about their sufficient financial sources (yes or no), housing type (purchased or leased), their own bedroom (yes or no), and pension (yes or no). Self-reported economic condition was also collected and categorized into very poor, poor, fair, rich, and very rich. To depict the comprehensive SES of each participant, the six economy-related variables were combined by principal component analyses to extract a synthesized variable. The overall SES variable was then classified into three tertiles, representing low SES, medium SES, and high SES.

### Health behavior assessment

We obtained five health behaviors: smoking, drinking, physical activity, sleep duration, and BMI. Self-reported smoking was divided into non-smoker (representing never smokers) and smoker (representing ex-smokers or current smokers) (22–24). Alcohol drinking was self-reported and grouped into non-drinker and drinker. Regular physical activity, referring to two or more purposeful fitness activities a week, was recorded as yes or no. Sleep duration, including nighttime and daytime sleep, was divided into four groups: ≤ 5.0 h, 5.1 − 7.0 h, 7.1 − 8.0 h, and > 8.0 h based on previous research (25). Height and weight were measured by the trained research assistants to calculate BMI as weight (kg) divided by square height (m^2^). Underweight (BMI < 18.5), normal (18.5 ≤ BMI < 24), overweight (24 ≤ BMI < 28), and obesity (BMI ≥ 28) were defined according to the Chinese BMI criteria (26).

Some sociodemographic variables at the baseline were also obtained, including marriage (in marriage and not in marriage), age groups (60–69, 70–79, and 80 and over years old), gender (male, female), and residence area (rural, town, and city).

### Multi-state model and statistical analyses

The baseline characteristics were presented as numbers (percentages) and compared across different SES levels by the Chi-squared tests. Cox regression model was firstly used to explore risk factors of first cardiometabolic disease, cardiometabolic multimorbidity, and mortality in separate models. Proportionality of hazards was assessed for each variable, and Schoenfeld residuals were visually inspected for potential time-variant biases. A *p*-value threshold of 0.05 was used to determine the significance in assessing the proportionality of hazards assumption and visual inspection of Schoenfeld residuals. Multi-state analyses were used to explore the effects of risk factors on transitions between the states.

**FIGURE 2:**
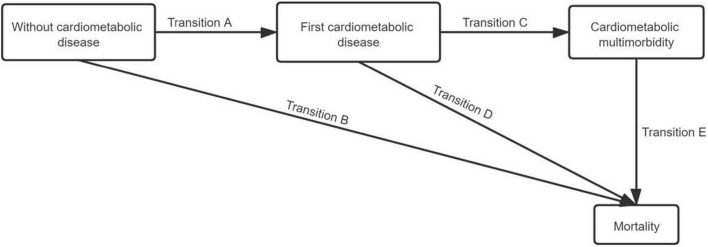
Schematic representation of the transitions between start of follow-up (without cardiometabolic disease), first cardiometabolic disease, cardiometabolic multimorbidity, and mortality.

In addition, the acyclic multi-state model shown in Figure 2 was used to define an interconnected progressive cardiometabolic multimorbidity system for the elders. In the system, four clinical states were determined: without cardiometabolic diseases (state 1), first cardiometabolic disease (state 2), cardiometabolic multimorbidity (state 3), and mortality (state 4). An individual began without cardiometabolic diseases (state 1) and moved toward the absorbing state to death (state 4) directly or through two different intermediates. Possible disease progression for participants included transition A (TA, transition from without cardiometabolic diseases into first cardiometabolic disease), transition B (TB, a direct transition from without cardiometabolic diseases into mortality), transition C (TC, participant developed the first cardiometabolic disease and subsequently cardiometabolic multimorbidity), transition D (TD, transition from cardiometabolic multimorbidity into mortality), and transition E (TE, transition from cardiometabolic multimorbidity into mortality).

Considering that intermediate events may change the natural history of the disease development, that is to say, the role of some risk factors may not be the same after the intermediate events, we fitted an illness-death model without recovery using the “mstate” package in R software (27) to explore factors’ effects during the disease progression. All subjects started in the without cardiometabolic diseases state, and some subjects remained in this state until the end of the study.

The robustness of our findings was checked using several sensitivity analyses. Firstly, we imputed the missing values of covariates using multiple imputation with a predictive mean matching method (28). Secondly, we repeated all analyses by calculating SES using latent class analyses (29). Thirdly, we excluded individuals with disability at the baseline because disability could influence both lifestyles and SES. Fourthly, we repeated cox regression and multi-state model analyses for participants with different genders and SES. The interactions were also tested by comparing models with and without a cross-product term between the risk factors and gender/SES for both cox and multi-state models. Fifthly, we excluded individuals with cardiometabolic diseases within 2 years after recruitment. Finally, we used age instead of the time since baseline as the time scale for the baseline hazards and tested whether age is more appropriate than the time since baseline. All statistical analyses in this study were performed using R version 3.3.2 (R Foundation for Statistical Computing, Vienna, Austria), and a two-sided *p* < 0.05 was considered statistically significant.

## Results

### Population characteristics

A total of 13,933 people in the CLHLS were included in this study from 2002 to 2014, with 73399.06 person-years of follow-up. Supplementary Table 1 shows the characteristics of the subjects by survey wave. Table 1 shows the baseline characteristics of the study population with cardiometabolic conditions and mortality status at the end of follow-up. Of the 13,933 participants, 6,016 (43.2%) were male, 9,418 (67.6%) resided in rural areas, 2,766 (19.9%) experienced first cardiometabolic disease, 975 (7.0%) met the criteria of multimorbidity, and 9,365 (67.2%) died over the follow-up period. The demographic characteristics and life behaviors were all consistently associated with the risk of the three outcomes. The SES indicators among the participants, such as self-assessed economic status, housing types, and adequate financial resources might influence the prevalence of mortality.

**TABLE 1:** Baseline characteristics of study population with cardiometabolic conditions and mortality status at the end of follow-up^a^.

Characteristics	First cardiometabolic disease			Cardiometabolic multimorbidity			Mortality		
			
	No (*n* = 11,167)	Yes (*n* = 2,766)	*P*-value^b^	No (*n* = 12,958)	Yes (*n* = 975)	*P*-value^b^	No (*n* = 4,568)	Yes (*n* = 9,365)	*P*-value^b^
***Demographic indicators***									
**Gender**									
Male	4,677 (41.9)	1,339 (48.4)	<0.001	5,546 (42.8)	470 (48.2)	0.001	2,216 (48.5)	3,800 (40.6)	<0.001
Female	6,490 (58.1)	1,427 (51.6)		7,412 (57.2)	505 (51.8)		2,352 (51.5)	5,565 (59.4)	
**Age (y)**									
60–69	1,340 (12.0)	820 (29.6)	<0.001	1,791 (13.8)	369 (37.8)	<0.001	1,660 (36.3)	500 (5.3)	<0.001
70–79	1,441 (12.9)	792 (28.6)		1,928 (14.9)	305 (31.3)		1,249 (27.3)	984 (10.5)	
≥80	8,386 (75.1)	1,154 (41.7)		9,239 (71.3)	301 (30.9)		1,659 (36.4)	7,881 (84.2)	
**Marriage**									
In marriage	3,378 (30.2)	1,546 (55.9)	<0.001	4,339 (33.5)	585 (60.0)	<0.001	2,696 (59.0)	2,228 (23.8)	<0.001
Not in marriage	7,789 (69.8)	1,220 (44.1)		8,619 (66.5)	390 (40.0)		1,872 (41.0)	7,137 (76.2)	
**Residence**									
Rural	7,580 (67.9)	1,838 (66.4)	0.096	8,798 (67.9)	620 (63.6)	0.003	2,916 (63.8)	6,502 (69.4)	<0.001
Town	1,998 (17.9)	544 (19.7)		2,359 (18.2)	183 (18.8)		861 (18.8)	1,681 (18.0)	
City	1,589 (14.2)	384 (13.9)		1,801 (13.9)	172 (17.6)		791 (17.4)	1,182 (12.6)	
***Socioeconomic indicators***									
**Education**									
No education	7,605 (68.1)	1,573 (56.9)	<0.001	8,671 (66.9)	507 (52.0)	<0.001	2,369 (51.9)	6,809 (72.7)	<0.001
Elementary school	2,222 (19.9)	682 (24.7)		2,659 (20.5)	245 (25.1)		1,156 (25.3)	1,748 (18.7)	
Secondary school	1,110 (9.9)	422 (15.3)		1,356 (10.5)	176 (18.1)		844 (18.5)	688 (7.3)	
University or above	230 (2.1)	89 (3.2)		272 (2.1)	47 (4.8)		199 (4.3)	120 (1.3)	
**Housing types**									
Purchased	10,508 (94.1)	2,594 (93.8)	0.528	12,199 (94.1)	903 (92.6)	0.052	4,232 (92.6)	8,870 (94.7)	<0.001
Leased	659 (5.9)	172 (6.2)		759 (5.9)	72 (7.4)		336 (7.4)	495 (5.3)	
**Have one’s own bedroom**									
Yes	10,170 (91.1)	2,521 (91.1)	0.907	11,808 (91.1)	883 (90.6)	0.553	4,167 (91.2)	8,524 (91.0)	0.695
No	997 (8.9)	245 (8.9)		1,150 (8.9)	92 (9.4)		401 (8.8)	841 (9.0)	
**Adequate financial resources**									
Yes	8,827 (79.0)	2,213 (80.0)	0.264	10,248 (79.1)	792 (81.2)	0.111	3,693 (80.8)	7,347 (78.5)	0.001
No	2,340 (21.0)	553 (20.0)		2,710 (20.9)	183 (18.8)		875 (19.2)	2,018 (21.5)	
**Self-assessed economic status**									
Very poor	320 (2.9)	58 (2.1)	0.140	366 (2.8)	12 (1.2)	0.001	93 (2.0)	285 (3.0)	<0.001
Poor	1,493 (13.4)	373 (13.5)		1,759 (13.6)	107 (11.0)		542 (11.9)	1,324 (14.1)	
Fair	7,647 (68.5)	1,885 (68.1)		8,853 (68.3)	679 (69.6)		3,158 (69.1)	6,374 (68.1)	
Rich	1,596 (14.3)	416 (15.0)		1,848 (14.3)	164 (16.8)		715 (15.7)	1,297 (13.8)	
Very rich	111 (1.0)	34 (1.2)		132 (1.0)	13 (1.3)		60 (1.3)	85 (0.9)	
**Pension**									
Yes	1,417 (12.7)	483 (17.5)	<0.001	1,636 (12.6)	264 (27.1)	<0.001	953 (20.9)	947 (10.1)	<0.001
No	9,750 (87.3)	2,283 (82.5)		11,322 (87.4)	711 (72.9)		3,615 (79.1)	8,418 (89.9)	
***Behavioral indicators***									
**Regular physical activity**									
Yes	2,625 (23.5)	858 (31.0)	<0.001	3,180 (24.5)	303 (31.1)	<0.001	1,425 (31.2)	2,058 (22.0)	<0.001
No	8,542 (76.5)	1,908 (69.0)		9,778 (75.5)	672 (68.9)		3,143 (68.8)	7,307 (78.0)	
**Smoking**									
Non-smoker	7,667 (68.7)	1,780 (64.4)	<0.001	8,823 (68.1)	624 (64.0)	0.008	2,973 (65.1)	6,474 (69.1)	<0.001
Smoker	3,500 (31.3)	986 (35.6)		4,135 (31.9)	351 (36.0)		1,595 (34.9)	2,891 (30.9)	
**Alcohol drinking**									
Non-drinker	7,826 (70.1)	1,892 (68.4)	0.085	9,058 (69.9)	660 (67.7)	0.147	3,128 (68.5)	6,590 (70.4)	0.022
Drinker	3,341 (29.9)	874 (31.6)		3,900 (30.1)	315 (32.3)		1,440 (31.5)	2,775 (29.6)	
**Sleep duration**									
≤5.0 h	1,213 (10.9)	319 (11.5)	<0.001	1,424 (11.0)	108 (11.1)	<0.001	509 (11.1)	1,023 (10.9)	<0.001
5.1–7.0 h	1,440 (12.9)	402 (14.5)		1,702 (13.1)	140 (14.4)		711 (15.6)	1,131 (12.1)	
7.1–8.0 h	4,091 (36.6)	1,212 (43.8)		4,837 (37.3)	466 (47.8)		2,077 (45.5)	3,226 (34.4)	
>8.0 h	4,423 (39.6)	833 (30.1)		4,995 (38.5)	261 (26.8)		1,271 (27.8)	3,985 (42.6)	
**BMI categories**									
Underweight	4,631 (41.5)	746 (27.0)	<0.001	5,187 (40.0)	190 (19.5)	<0.001	1,156 (25.3)	4,221 (45.1)	<0.001
Normal	5,470 (49.0)	1,581 (57.2)		6,490 (50.1)	561 (57.5)		2,655 (58.1)	4,396 (46.9)	
Overweight	850 (7.6)	364 (13.2)		1,038 (8.0)	176 (18.1)		608 (13.3)	606 (6.5)	
Obesity	216 (1.9)	75 (2.7)		243 (1.9)	48 (4.9)		149 (3.3)	142 (1.5)	

SES, socioeconomic status; BMI, body mass index (calculated as weight in kilograms divided by square of height in meters).

^a^Data are presented as number (percentage) unless otherwise indicated. ^b^The χ^2^ test was used for categorical variables.

### Cox regression analyses

In the Cox regression, demographic indicators were significantly associated with the odds of the three outcome variables, as shown in Table 2. For example, city residents were more likely to suffer from adverse outcomes. Higher age was an important risk factor for the first cardiometabolic disease and mortality. Especially, higher than 80 years had the strongest association with mortality (HR = 7.83, 95% CI, 7.1 − 8.65). Participants who were not in marriage had a higher risk of mortality than their married counterparts (HR = 1.59, 95% CI, 1.51 − 1.68). The difference in mortality between genders indicated that females had longer life expectancy than males.

**TABLE 2:** Association of factors with cardiometabolic conditions and mortality using Cox regression model.

Factors	First cardiometabolic disease		Cardiometabolic multimorbidity		Mortality	
			
	*n*	HR (95%CI)	*n*	HR (95%CI)	*N*	HR (95%CI)
**Gender**						
Male	1,339	1.00 [Ref.]	470	1.00 [Ref.]	3,800	1.00 [Ref.]
Female	1,427	0.99 (0.90–1.08)	505	1.08 (0.92–1.27)	5,565	**0.89 (0.85–0.94)**
**Age (y)**						
60–69	820	1.00 [Ref.]	369	1.00 [Ref.]	500	1.00 [Ref.]
70–79	792	**1.18 (1.07–1.31)**	305	1.09 (0.94–1.28)	984	**2.02 (1.81–2.25)**
≥80	1,154	**1.26 (1.13–1.40)**	301	1.02 (0.86–1.22)	7,881	**7.83 (7.10–8.65)**
**Marriage**						
In marriage	1,546	1.00 [Ref.]	585	1.00 [Ref.]	2,228	1.00 [Ref.]
Not in marriage	1,220	0.94 (0.86–1.03)	390	1.04 (0.89–1.21)	7,137	**1.59 (1.51–1.68)**
**Residence**						
Rural	1,838	1.00 [Ref.]	620	1.00 [Ref.]	6,502	1.00 [Ref.]
Town	544	1.07 (0.97–1.18)	183	1.03 (0.87–1.22)	1,681	1.01 (0.96–1.07)
City	384	**1.20 (1.07–1.34)**	172	**1.48 (1.24–1.78)**	1,182	**1.12 (1.05–1.19)**
**SES**						
Low SES	878	1.00 [Ref.]	291	1.00 [Ref.]	3,218	1.00 [Ref.]
Medium SES	1,121	1.00 (0.91–1.11)	341	0.87 (0.73–1.04)	4,153	0.99 (0.94–1.04)
High SES	781	0.92 (0.84–1.01)	345	1.05 (0.90–1.23)	2,043	**0.91 (0.87–0.96)**
**Regular physical activity**						
Yes	858	1.00 [Ref.]	303	1.00 [Ref.]	2,058	1.00 [Ref.]
No	1,908	0.94 (0.87–1.03)	672	1.14 (0.99–1.32)	7,307	**1.24 (1.18–1.31)**
**Smoking**						
Non-smoker	1,780	1.00 [Ref.]	624	1.00 [Ref.]	6,474	1.00 [Ref.]
Smoker	986	1.03 (0.93–1.14)	351	0.99 (0.83–1.17)	2,891	**1.08 (1.02–1.15)**
**Alcohol drinking**						
Non-drinker	1,892	1.00 [Ref.]	660	1.00 [Ref.]	6,590	1.00 [Ref.]
Drinker	874	0.94 (0.85–1.04)	315	1.00 (0.85–1.18)	2,775	1.04 (0.98–1.10)
**Sleep duration**						
≤5.0 h	319	1.04 (0.92–1.18)	108	0.95 (0.77–1.18)	1,023	1.04 (0.97–1.12)
5.1–7.0 h	402	1.01 (0.90–1.13)	140	0.93 (0.77–1.12)	1,131	1.00 (0.93–1.07)
7.1–8.0 h	1,212	1.00 [Ref.]	466	1.00 [Ref.]	3,226	1.00 [Ref.]
>8.0 h	833	0.98 (0.90–1.08)	261	0.89 (0.76–1.03)	3,985	**1.20 (1.15–1.26)**
**BMI categories**						
Underweight	746	**0.87 (0.79–0.95)**	190	**0.69 (0.59–0.82)**	4,221	**1.22 (1.17–1.27)**
Normal	1,581	1.00 [Ref.]	561	1.00 [Ref.]	4,396	1.00 [Ref.]
Overweight	364	**1.33 (1.19–1.50)**	176	**1.63 (1.37–1.94)**	606	**0.86 (0.79–0.94)**
Obesity	75	**1.28 (1.01–1.61)**	48	**2.02 (1.50–2.72)**	142	1.00 (0.85–1.19)

SES, socioeconomic status; BMI, body mass index (calculated as weight in kilograms divided by square of height in meters); HR, hazard ratio. Bold values indicate that there are statistically significant differences.

We further analyzed the association of SES with the three outcomes. However, it was only found that higher SES might decrease the risk for mortality (HR = 0.91, 95% CI, 0.87 − 0.96). Among the behavioral risk factors, overweight/obesity had a robust positive association with the incidence of first cardiometabolic disease, cardiometabolic multimorbidity, and mortality. Other behavioral risk factors such as physical inactivity (HR = 1.24, 95% CI, 1.18 − 1.31), smokers (HR = 1.08, 95% CI, 1.02 − 1.15), and > 8 h of sleep duration (HR = 1.20, 95% CI, 1.15 − 1.26) were all more likely to increase the risk for mortality but were not associated with the incidence of first cardiometabolic disease and cardiometabolic multimorbidity.

### Multi-state model analyses

The role of risk factors in the transitions from healthy to multimorbidity and mortality is shown in Table 3. We examined the associations of individual demographic, SES, and behavioral risk factors with the transitions and found that advanced age was the only risk factor for all four transitions except for TC. Although females had a higher risk for first cardiometabolic disease (TA) and multimorbidity (TC), they had a lower incidence of mortality (TB and TD) than the males. TA and TC were more likely to occur among city residents, but the probability of transition E was lower. Participants who were not in marriage had a higher risk for TB and TD. Higher SES was associated with a higher risk for TA and a lower risk for TB among the healthy participants.

**TABLE 3:** Role of factors in transitions between cardiometabolic conditions and mortality.

Factors	HR (95% CI)				
	
	A (healthy→ first disease)	B (healthy→ mortality)	C (first disease→ multimorbidity)	D (first disease→ mortality)	E (multimorbidity→ mortality)
**Gender**					
Male	1.00 [Ref.]	1.00 [Ref.]	1.00 [Ref.]	1.00 [Ref.]	1.00 [Ref.]
Female	**1.26 (1.15–1.39)**	**0.84 (0.80–0.89)**	**1.42 (1.10–1.85)**	**0.84 (0.73–0.97)**	1.25 (0.73–2.15)
**Age (y)**					
60–69	1.00 [Ref.]	1.00 [Ref.]	1.00 [Ref.]	1.00 [Ref.]	1.00 [Ref.]
70–79	**1.31 (1.18–1.44)**	**1.41 (1.26–1.57)**	**0.76 (0.59–0.98)**	**2.18 (1.81–2.62)**	**2.34 (1.43–3.81)**
≥80	1.08 (0.97–1.21)	**6.00 (5.48–6.57)**	**0.41 (0.29–0.59)**	**5.75 (4.84–6.83)**	**4.40 (2.33–8.33)**
**Marriage**					
In marriage	1.00 [Ref.]	1.00 [Ref.]	1.00 [Ref.]	1.00 [Ref.]	1.00 [Ref.]
Not in marriage	**0.85 (0.77–0.94)**	**1.64 (1.54–1.74)**	0.99 (0.75–1.29)	**1.24 (1.08–1.43)**	1.13 (0.68–1.89)
**Residence**					
Rural	1.00 [Ref.]	1.00 [Ref.]	1.00 [Ref.]	1.00 [Ref.]	1.00 [Ref.]
Town	**1.20 (1.09–1.32)**	0.99 (0.93–1.05)	1.07 (0.81–1.41)	0.99 (0.85–1.15)	0.98 (0.58–1.65)
City	**1.33 (1.19–1.50)**	**1.12 (1.05–1.20)**	**1.41 (1.04–1.93)**	0.93 (0.76–1.13)	**0.43 (0.21–0.88)**
**SES**					
Low SES	1.00 [Ref.]	1.00 [Ref.]	1.00 [Ref.]	1.00 [Ref.]	1.00 [Ref.]
Medium SES	**1.15 (1.04–1.27)**	**0.94 (0.89–0.99)**	0.76 (0.56–1.03)	1.03 (0.89–1.20)	1.26 (0.71–2.24)
High SES	**1.17 (1.07–1.28)**	**0.85 (0.81–0.90)**	1.08 (0.85–1.37)	0.87 (0.76–1.00)	1.27 (0.79–2.04)
**Regular physical activity**					
Yes	1.00 [Ref.]	1.00 [Ref.]	1.00 [Ref.]	1.00 [Ref.]	1.00 [Ref.]
No	**1.13 (1.04–1.23)**	**1.18 (1.12–1.24)**	1.11 (0.89–1.40)	1.11 (0.98–1.27)	0.79 (0.50–1.27)
**Smoking**					
Non-smoker	1.00 [Ref.]	1.00 [Ref.]	1.00 [Ref.]	1.00 [Ref.]	1.00 [Ref.]
Smoker	**1.20 (1.08–1.33)**	1.02 (0.96–1.09)	1.06 (0.80–1.42)	1.01 (0.86–1.18)	**2.25 (1.22–4.15)**
**Alcohol drinking**					
Non-drinker	1.00 [Ref.]	1.00 [Ref.]	1.00 [Ref.]	1.00 [Ref.]	1.00 [Ref.]
Drinker	1.01 (0.91–1.12)	1.01 (0.95–1.07)	0.87 (0.65–1.16)	1.06 (0.91–1.23)	1.10 (0.62–1.97)
**Sleep duration**					
≤5.0 h	**1.15 (1.02–1.30)**	0.98 (0.91–1.06)	0.85 (0.59–1.23)	1.17 (0.96–1.42)	0.80 (0.37–1.73)
5.1–7.0 h	**1.15 (1.02–1.28)**	0.94 (0.88–1.02)	0.83 (0.59–1.16)	1.05 (0.87–1.27)	1.15 (0.63–2.12)
7.1–8.0 h	1.00 [Ref.]	1.00 [Ref.]	1.00 [Ref.]	1.00 [Ref.]	1.00 [Ref.]
>8.0 h	1.04 (0.95–1.14)	**1.15 (1.10–1.21)**	0.87 (0.66–1.14)	**1.24 (1.09–1.43)**	1.05 (0.64–1.74)
**BMI categories**					
Underweight	**0.87 (0.80–0.95)**	**1.19 (1.14–1.25)**	0.75 (0.54–1.03)	**1.20 (1.05–1.37)**	1.21 (0.67–2.18)
Normal	1.00 [Ref.]	1.00 [Ref.]	1.00 [Ref.]	1.00 [Ref.]	1.00 [Ref.]
Overweight	**1.48 (1.32–1.66)**	**0.81 (0.73–0.89)**	**1.43 (1.08–1.90)**	0.93 (0.76–1.15)	1.21 (0.69–2.13)
Obesity	**1.34 (1.06–1.69)**	1.10 (0.92–1.31)	**1.75 (1.03–2.98)**	**0.52 (0.31–0.89)**	1.17 (0.35–3.90)

SES, socioeconomic status; BMI, body mass index (calculated as weight in kilograms divided by square of height in meters); HR, hazard ratio. Bold values indicate that there are statistically significant differences.

Behavior risk factors like physical inactivity, smokers, and longer or shorter sleep duration were all likely to increase the risk for first cardiometabolic disease and mortality among the healthy participants. Overweight and obesity were risk factors for developing into first cardiometabolic disease and then transitioning into multimorbidity. Furthermore, overweight and obesity were associated with a decreased hazard of mortality among participants with event-free state and first cardiometabolic disease, respectively.

Supplementary Tables show the results of sensitivity analyses. Before and after imputation, the differences in the baseline characteristics were not statistically significant (Supplementary Table 2). After using multiple imputations to impute missing values of all covariates, we repeated all analyses and observed similar findings (Supplementary Table 3). We used a new SES indicator based on latent class analyses to repeat all analyses in the multi-state model (Supplementary Table 4), and the results were broadly consistent with our main findings (Supplementary Table 5). After excluding participants with disability at the baseline, the results remained similar to the main findings (Supplementary Table 6). Moreover, the results showed several statistically significant interactions among participants after being stratified by gender and SES. Nevertheless, most of them seemed clinically meaningless (Supplementary Tables 7–16). After excluding cardiometabolic disease occurring within 2 years after recruitment, the results of multi-state models remained consistent in general (Supplementary Table 17). Analysis using the time since baseline with age as the time scale for the baseline hazards found unchangeable results. Thus, the time since baseline was as appropriate as the age for the baseline hazards (Supplementary Table 18).

## Discussion

To our best knowledge, this study is the first to examine cardiometabolic multimorbidity progression based on a large-scale, longitudinal cohort of older adults in LMICs, China. The multi-state model and Cox model were adopted simultaneously to investigate the role of demographic, socioeconomic, and behavioral factors on cardiometabolic progression. Two key findings were presented. First, sociodemographic characteristics, such as female, age, not in marriage, and urban residents, are important risk factors for first cardiometabolic disease, multimorbidity, and mortality. Especially, age is the strongest predictor of mortality in those with cardiometabolic multimorbidity and without cardiometabolic multimorbidity. Higher SES increases the incidence of first cardiometabolic disease and decreases cardiometabolic multimorbidity, which is contrary to the results in high-income countries. Second, behavioral indicators like smoking, physical inactivity, overweight/obesity, and non-optimal sleep time are associated with a higher risk for cardiometabolic progression and mortality.

Age is important for almost all transitions in the defined progressive cardiometabolic multimorbidity system. A higher age corresponds to a higher risk for first cardiometabolic disease and mortality. Previous studies have shown that aging leads to rapid increases in the prevalence of cardiometabolic diseases (6, 17). However, the risk of cardiometabolic multimorbidity decreases with age, which agrees with previous studies. Yao et al. have found that the prevalence of multimorbidity sharply increases with age and then slightly decreases among the oldest-old adults (11). Abebe et al. have reported that the prevalence of multimorbidity rises in the population at the age around 40 years and then flattens population at the age over 70 years (8). The possible reasons are as follows. Firstly, the cognitive function of the elderly may be impaired, which means that there is a recall bias in the collected information, resulting in a low reporting rate of chronic diseases. Secondly, it is possible that the older adults who survived to the oldest-old but had fewer chronic diseases were enrolled. Thirdly, the compression of morbidity proposed by Andersen is also one possible explanation. With the population aging, the hazard of age-related diseases and overall morbidity becomes progressively less (30). Compared to males, females are more likely to develop cardiometabolic conditions but have a lower risk for mortality, partly attributed to their longer life expectancy and higher health awareness (31–33). Higher risk for TB and TD are observed among the participants who are not in marriage, possibly because married individuals might enjoy a healthier lifestyle, which provides long-term benefits for wellbeing (34, 35). However, individuals without a partner lack social support and are more likely to suffer adverse health outcomes. Moreover, urban residents have a higher risk of multimorbidity than rural residents. Generally, our results are consistent with most previous studies on the roles of demographic factors on cardiometabolic conditions and mortality (33, 35).

Our results revealed a statistically significant socioeconomic gradient in that the risk of first cardiometabolic disease increases with the SES level. Once a cardiometabolic disease is diagnosed, affluent participants have the ability to pay for ongoing treatment, which would improve their health literacy. Participants with high SES have greater access to health-care services. Thus, their non-communicable diseases are more likely to be diagnosed or even over-diagnosed than participants with low SES (36, 37). Because of the low accessibility of health facilities and poor health literacy among deprived individuals, cardiometabolic diseases might be under-reported and neglected, resulting in higher mortality risk (38–41). Low healthcare-seeking behavior and the probability of underdiagnoses might have contributed to the increased mortality of people with low SES in China.

Our finding also highlights that adverse behavior factors, such as physical inactivity, smoking, and longer or shorter sleep duration, are likely to increase the risk for first cardiometabolic disease and mortality in healthy participants. It is globally known that healthy lifestyles decrease the risk of cardiometabolic conditions and mortality and increase life expectancy (31, 42), particularly in those without cardiometabolic conditions. Both experimental and epidemiological studies have shown that the decline in sleep time and sleep quality is a risk factor for cardiometabolic abnormalities (43, 44), consistent with our results. Previous studies have identified a “U” or “J” relationship between sleep time and all-cause death. A “U”-shaped association represents that longer or shorter sleep is associated with an increased risk of death (45). However, our present study found that only older adults who sleep longer have a higher mortality risk (46). Overweight or obese participants tend to develop into first cardiometabolic disease and then transfer into multimorbidity in contrast with participants with normal weight. Studies have suggested that obese participants have a higher risk for any CVD than those with normal weight (47–49). It has been inferred that obesity could cause dyslipidemia, systemic inflammation, and a series of metabolic abnormalities, which in turn increase the risk of vascular diseases and diabetes (50).

The two key findings in the present study have important implications for preventing and controlling cardiometabolic diseases. Firstly, some sociodemographic characteristics, such as being female, age, not in marriage, and residency, contribute to the occurrence of the first cardiometabolic disease, multimorbidity, and mortality. High SES plays an important role in increasing the risk for the first cardiometabolic disease and decreasing the risk for cardiometabolic multimorbidity. Previous studies have also found that socioeconomic factors are associated with multimorbidity. However, most of these studies are confined to a unique outcome rather than the progression from the first cardiometabolic disease to multimorbidity and death within a single analytic framework (1, 15–17). The importance of sociodemographic characteristics for the incidence of the first cardiometabolic disease, multimorbidity, and mortality suggests that basic sociodemographic factors should be targeted sufficiently in primary prevention. To minimize transition risks for cardiometabolic morbidity and mortality, policymakers also need to evaluate these facts from a financial perspective, providing and implementing sufficient required health preventive strategies, especially for those with low SES. Secondly, our results indicate that adverse behavior factors promote the development of the first disease and mortality in the healthy participants. A previous study has shown that midlife behavioral factors increase mortality among participants with cardiometabolic diseases (51). The CHANCES study confirmed that at the age of 50 years, a healthy lifestyle increases life expectancy by 7.4 − 15.7 years and leads to free of chronic diseases in most cases (52). Li et al. have found that a low-risk lifestyle will prevent 90% of diabetes, 80% of coronary heart diseases, and 70% of cardiovascular mortality in the United States (53). However, the evidence on the effective measures to treat patients with several medical conditions is limited in LMICs, in which persons may face accumulating and overwhelming complexity due to the uncoordinated responses to the problems. Thus, our results could provide the priority directed to reorienting and strengthening the health care system in tackling this challenge in LMICs. Our study has several strengths. Firstly, the study was derived from large samples in China. Secondly, the long-term follow-up allowed us to obtain complete data on health outcomes and analyze clinically diagnosed incident diseases. Thirdly, the overall SES variable established in this study empowered us to comprehensively evaluate the complex relationship of SES with mortality and with the progression of cardiometabolic conditions. Fourthly, the robustness of our findings was further evaluated using a series of sensitivity analyses. Fifthly, the role of risk factors in the progression from the first cardiometabolic disease to multimorbidity and death was evaluated within a single analytic framework and using conventional survival analyses.

Nevertheless, our study has several limitations. Firstly, data used in this study were collected every 2–3 years. Therefore, some transitions might be missed during the long interval between measurements. Secondly, all risk factors were assessed at the baseline; changes in risk factor levels due to the onset of disease or lifestyle modification were not examined. Thirdly, although the key personal characteristics and lifestyle behaviors were considered during the analyses, other potential confounders were not excluded. Furthermore, the study was observational. Therefore, a certain causal relationship cannot be demonstrated. Finally, to simplify the analyses and interpretation, the multi-state model only included five transitions rather than all possible transitions between individual diseases and outcomes and between pairs of diseases. Beyond that, data about the cardiometabolic diseases were self-reported based on the subject’s memory, which might be flawed.

## Conclusion

Our analyses presented potential risk factors for individual cardiometabolic disease transitions. Behavioral factors, such as smoking, physical inactivity, overweight/obesity, and non-optimal sleep duration are the key determinants of cardiometabolic progression and mortality. Higher SES is associated with a greater prevalence of the first cardiometabolic disease and a lower risk for mortality among healthy participants. These findings highlight the importance of lifestyle and SES modifications in reducing disease burden and have important implications for policy-makers to address the upstream social and behavioral determinants of health.

## Data availability statement

The data presented in this study are deposited in the CLHLS repository, accession link https://opendata.pku.edu.cn/dataverse/CHADS. Our data used in this study has been uploaded as Supplementary materials.

## Ethics statement

The studies involving human participants were reviewed and approved by the Research Ethics Committees of Duke University and Peking University (IRB00001052-13074). The participants provided their informed consent and research ethics approval to participate in this study.

## Author contributions

LP and HZ conceived the study and drafted the manuscript. HZ, XD, PR, YD, and MY contributed to the acquisition, analyses, and interpretation of data. HZ, LP, YZ, FC, YC, JZ, and DW participated in critical revision of the manuscript for important intellectual content. All authors contributed to the critical revisions and final approval of the manuscript.

## Footnotes

1
http://opendata.pku.edu.cn/

## References

[1] Zhang D Tang X Shen P Si Y Liu X Xu Z Multimorbidity of cardiometabolic diseases: prevalence and risk for mortality from one million Chinese adults in a longitudinal cohort study. *BMJ Open.* (2019) 9:e024476. 10.1136/bmjopen-2018-024476 30833320 PMC6443196

[2] Arnett DK Blumenthal RS Albert MA Buroker AB Goldberger ZD Hahn EJ 2019 ACC/AHA guideline on the primary prevention of cardiovascular disease: a report of the American College of Cardiology/American Heart Association task force on clinical practice guidelines. *Circulation.* (2019) 140:e596–646. 10.1161/CIR.0000000000000678 30879355 PMC7734661

[3] Ralston J Nugent R. Toward a broader response to cardiometabolic disease. *Nat Med.* (2019) 25:1644–6. 10.1038/s41591-019-0642-9 31700172

[4] Palladino R Tayu Lee J Ashworth M Triassi M Millett C. Associations between multimorbidity, healthcare utilisation and health status: evidence from 16 European countries. *Age Ageing.* (2016) 45:431–5. 10.1093/ageing/afw044 27013499 PMC4846796

[5] Emerging Risk Factors Collaboration, Di Angelantonio E Kaptoge S Wormser D Willeit P Butterworth AS Association of cardiometabolic multimorbidity with mortality. *JAMA.* (2015) 314:52–60. 10.1001/jama.2015.7008 26151266 PMC4664176

[6] Lyall DM Celis-Morales CA Anderson J Gill JM Mackay DF McIntosh AM Associations between single and multiple cardiometabolic diseases and cognitive abilities in 474 129 UK Biobank participants. *Eur Heart J.* (2017) 38:577–83. 10.1093/eurheartj/ehw528 28363219 PMC5381595

[7] Violan C Foguet-Boreu Q Flores-Mateo G Salisbury C Blom J Freitag M Prevalence, determinants and patterns of multimorbidity in primary care: a systematic review of observational studies. *PLoS One.* (2014) 9:e102149. 10.1371/journal.pone.0102149 25048354 PMC4105594

[8] Abebe F Schneider M Asrat B Ambaw F. Multimorbidity of chronic non-communicable diseases in low- and middle-income countries: a scoping review. *J Comorb.* (2020) 10:2235042X20961919. 10.1177/2235042X20961919 33117722 PMC7573723

[9] Huaquía-Díaz AM Chalán-Dávila TS Carrillo-Larco RM Bernabe-Ortiz A. Multimorbidity in Latin America and the Caribbean: a systematic review and meta-analysis. *BMJ Open.* (2021) 11:e050409. 10.1136/bmjopen-2021-050409 34301665 PMC8311299

[10] Nguyen H Manolova G Daskalopoulou C Vitoratou S Prince M Prina AM. Prevalence of multimorbidity in community settings: a systematic review and meta-analysis of observational studies. *J Comorb.* (2019) 9:2235042X19870934. 10.1177/2235042X19870934 31489279 PMC6710708

[11] Yao SS Cao GY Han L Chen ZS Huang ZT Gong P Prevalence and patterns of multimorbidity in a nationally representative sample of older Chinese: results from the china health and retirement longitudinal study. *J Gerontol A Biol Sci Med Sci.* (2020) 75:1974–80. 10.1093/gerona/glz185 31406983

[12] Basto-Abreu A Barrientos-Gutierrez T Wade AN Oliveira de Melo D Semeão de Souza AS Nunes BP. Multimorbidity matters in low and middle-income countries. *J Multimorb Comorb.* (2022) 12:26335565221106074. 10.1177/26335565221106074 35734547 PMC9208045

[13] GBD 2017 DALYs and Hale Collaborators. Global, regional, and national disability-adjusted life-years (DALYs) for 359 diseases and injuries and healthy life expectancy (HALE) for 195 countries and territories, 1990-2017: a systematic analysis for the Global Burden of Disease Study 2017. *Lancet.* (2018) 392:1859–922. 10.1016/S0140-6736(18)32335-3 30415748 PMC6252083

[14] Tinetti ME Fried TR Boyd CM. Designing health care for the most common chronic condition–multimorbidity. *JAMA.* (2012) 307:2493–4. 10.1001/jama.2012.5265 22797447 PMC4083627

[15] Kivimäki M Kuosma E Ferrie JE Luukkonen R Nyberg ST Alfredsson L Overweight, obesity, and risk of cardiometabolic multimorbidity: pooled analysis of individual-level data for 120 813 adults from 16 cohort studies from the USA and Europe. *Lancet Public Health.* (2017) 2:e277–85. 10.1016/S2468-2667(17)30074-9 28626830 PMC5463032

[16] Feng L Jehan I de Silva HA Naheed A Farazdaq H Hirani S Prevalence and correlates of cardiometabolic multimorbidity among hypertensive individuals: a cross-sectional study in rural South Asia- Bangladesh, Pakistan and Sri Lanka. *BMJ Open.* (2019) 9:e030584. 10.1136/bmjopen-2019-030584 31488490 PMC6731877

[17] Fortin M Haggerty J Almirall J Bouhali T Sasseville M Lemieux M. Lifestyle factors and multimorbidity: a cross sectional study. *BMC Public Health.* (2014) 14:686. 10.1186/1471-2458-14-686 24996220 PMC4096542

[18] Singh-Manoux A Fayosse A Sabia S Tabak A Shipley M Dugravot A Clinical, socioeconomic, and behavioural factors at age 50 years and risk of cardiometabolic multimorbidity and mortality: a cohort study. *PLoS Med.* (2018) 15:e1002571. 10.1371/journal.pmed.1002571 29782486 PMC5962054

[19] Peking University Open Research Data. *Center for Health Aging and Development.* (2021). Available online at: https://opendata.pku.edu.cn/dataverse/CHADS (accessed Aug 31, 2021).

[20] Zhang Q Wu Y Han T Liu E. Changes in cognitive function and risk factors for cognitive impairment of the elderly in China: 2005-2014. *Int J Environ Res Public Health.* (2019) 16:2847. 10.3390/ijerph16162847 31404951 PMC6719934

[21] Li J Wu B Tevik K Krokstad S Helvik AS. Factors associated with elevated consumption of alcohol in older adults-comparison between China and Norway: the CLHLS and the HUNT study. *BMJ Open.* (2019) 9:e028646. 10.1136/bmjopen-2018-028646 31377703 PMC6687031

[22] Liu E Feng Y Yue Z Zhang Q Han T. Differences in the health behaviors of elderly individuals and influencing factors: evidence from the Chinese Longitudinal Healthy Longevity Survey. *Int J Health Plann Manage.* (2019) 34:e1520–32. 10.1002/hpm.2824 31149759

[23] Shi X Lv Y Mao C Yuan J Yin Z Gao X Garlic consumption and all-cause mortality among Chinese oldest-old individuals: a population-based cohort study. *Nutrients.* (2019) 11:1504. 10.3390/nu11071504 31262080 PMC6683033

[24] Lv X Li W Ma Y Chen H Zeng Y Yu X Cognitive decline and mortality among community-dwelling Chinese older people. *BMC Med.* (2019) 17:63. 10.1186/s12916-019-1295-8 30871536 PMC6419492

[25] Jackowska M Poole L. Sleep problems, short sleep and a combination of both increase the risk of depressive symptoms in older people: a 6-year follow-up investigation from the English Longitudinal Study of Ageing. *Sleep Med.* (2017) 37:60–5. 10.1016/j.sleep.2017.02.004 28899541

[26] Zhou BF Cooperative Meta-Analysis Group of the Working Group on Obesity in China. Predictive values of body mass index and waist circumference for risk factors of certain related diseases in Chinese adults-study on optimal cut-off points of body mass index and waist circumference in Chinese adults. *Biomed Environ Sci.* (2002) 15:83–96. 12046553

[27] de Wreede LC Fiocco M Putter H. The mstate package for estimation and prediction in non- and semi-parametric multi-state and competing risks models. *Comput Methods Programs Biomed.* (2010) 99:261–74. 10.1016/j.cmpb.2010.01.001 20227129

[28] Austin PC White IR Lee DS van Buren S. Missing data in clinical research: a tutorial on multiple imputation. *Can J Cardiol.* (2021) 37:1322–31. 10.1016/j.cjca.2020.11.010 33276049 PMC8499698

[29] Zhang YB Chen C Pan XF Guo J Li Y Franco OH Associations of healthy lifestyle and socioeconomic status with mortality and incident cardiovascular disease: two prospective cohort studies. *BMJ.* (2021) 373:n604. 10.1136/bmj.n604 33853828 PMC8044922

[30] Andersen SL Sebastiani P Dworkis DA Feldman L Perls TT. Health span approximates life span among many supercentenarians: compression of morbidity at the approximate limit of life span. *J Gerontol A Biol Sci Med Sci.* (2012) 67:395–405. 10.1093/gerona/glr223 22219514 PMC3309876

[31] Chudasama YV Khunti K Gillies CL Dhalwani NN Davies MJ Yates T Healthy lifestyle and life expectancy in people with multimorbidity in the UK Biobank: a longitudinal cohort study. *PLoS Med.* (2020) 17:e1003332. 10.1371/journal.pmed.1003332 32960883 PMC7508366

[32] Nyberg ST Singh-Manoux A Pentti J Madsen IEH Sabia S Alfredsson L Association of healthy lifestyle with years lived without major chronic diseases. *JAMA Intern Med.* (2020) 180:760–8. 10.1001/jamainternmed.2020.0618 32250383 PMC7136858

[33] Jani BD Hanlon P Nicholl BI McQueenie R Gallacher KI Lee D Relationship between multimorbidity, demographic factors and mortality: findings from the UK Biobank cohort. *BMC Med.* (2019) 17:74. 10.1186/s12916-019-1305-x 30967141 PMC6456941

[34] Lopes JM Galvão FD Oliveira AGRDC. Risk of death in the elderly with excessive daytime sleepiness, insomnia and depression: prospective cohort study in an urban population in Northeast Brazil. *Arq Bras Cardiol.* (2021) 117:446–54. 10.36660/abc.20200059 34161418 PMC8462963

[35] Zueras P Rutigliano R Trias-Llimós S. Marital status, living arrangements, and mortality in middle and older age in Europe. *Int J Public Health.* (2020) 65:627–36. 10.1007/s00038-020-01371-w 32350551 PMC7360666

[36] Zhao Y Atun R Oldenburg B McPake B Tang S Mercer SW Physical multimorbidity, health service use, and catastrophic health expenditure by socioeconomic groups in China: an analysis of population-based panel data. *Lancet Glob Health.* (2020) 8:e840–9. 10.1016/S2214-109X(20)30127-3 32446349 PMC7241981

[37] Pati S Agrawal S Swain S Lee JT Vellakkal S Hussain MA Non communicable disease multimorbidity and associated health care utilization and expenditures in India: cross-sectional study. *BMC Health Serv Res.* (2014) 14:451. 10.1186/1472-6963-14-451 25274447 PMC4283077

[38] Stoner L Matheson AG Perry LG Williams MA McManus A Holdaway M Social contributors to cardiometabolic diseases in indigenous populations: an international Delphi study. *Public Health.* (2019) 176:133–41. 10.1016/j.puhe.2018.08.012 31796166

[39] Pearson AL Bentham G Day P Kingham S. Associations between neighbourhood environmental characteristics and obesity and related behaviours among adult New Zealanders. *BMC Public Health.* (2014) 14:553. 10.1186/1471-2458-14-553 24894572 PMC4059100

[40] Chetty R Stepner M Abraham S Lin S Scuderi B Turner N The association between income and life expectancy in the United States, 2001-2014. *JAMA.* (2016) 315:1750–66. 10.1001/jama.2016.4226 27063997 PMC4866586

[41] Stringhini S Carmeli C Jokela M Avendaño M Muennig P Guida F Socioeconomic status and the 25 × 25 risk factors as determinants of premature mortality: a multicohort study and meta-analysis of 1⋅7 million men and women. *Lancet.* (2017) 389:1229–37. 10.1016/S0140-6736(16)32380-7 28159391 PMC5368415

[42] Xu X Mishra GD Dobson AJ Jones M. Progression of diabetes, heart disease, and stroke multimorbidity in middle-aged women: a 20-year cohort study. *PLoS Med.* (2018) 15:e1002516. 10.1371/journal.pmed.1002516 29534066 PMC5849280

[43] Farr OM Mantzoros CS. Advances at the intersection of sleep and metabolism research. *Metabolism.* (2018) 84:1–2. 10.1016/j.metabol.2018.03.026 29634954

[44] Sakamoto N Gozal D Smith DL Yang L Morimoto N Wada H Sleep duration, snoring prevalence, obesity, and behavioral problems in a large cohort of primary school students in Japan. *Sleep.* (2017) 40:zsw082. 10.1093/sleep/zsw082 28364432

[45] Yin J Jin X Shan Z Li S Huang H Li P Relationship of sleep duration with all-cause mortality and cardiovascular events: a systematic review and dose-response meta-analysis of prospective cohort studies. *J Am Heart Assoc.* (2017) 6:e005947. 10.1161/JAHA.117.005947 28889101 PMC5634263

[46] García-Perdomo HA Zapata-Copete J Rojas-Cerón CA. Sleep duration and risk of all-cause mortality: a systematic review and meta-analysis. *Epidemiol Psychiatr Sci.* (2019) 28:578–88. 10.1017/S2045796018000379 30058510 PMC6998920

[47] Doustmohamadian S Serahati S Barzin M Keihani S Azizi F Hosseinpanah F. Risk of all-cause mortality in abdominal obesity phenotypes: tehran lipid and glucose study. *Nutr Metab Cardiovasc Dis.* (2017) 27:241–8. 10.1016/j.numecd.2016.11.123 28139376

[48] Mongraw-Chaffin ML Peters SAE Huxley RR Woodward M. The sex-specific association between BMI and coronary heart disease: a systematic review and meta-analysis of 95 cohorts with 1⋅2 million participants. *Lancet Diabetes Endocrinol.* (2015) 3:437–49. 10.1016/S2213-8587(15)00086-8 25960160 PMC4470268

[49] Keihani S Hosseinpanah F Barzin M Serahati S Doustmohamadian S Azizi F. Abdominal obesity phenotypes and risk of cardiovascular disease in a decade of follow-up: the Tehran lipid and glucose study. *Atherosclerosis.* (2015) 238:256–63. 10.1016/j.atherosclerosis.2014.12.008 25540856

[50] Bell JA Hamer M Batty GD Singh-Manoux A Sabia S Kivimäki M. Incidence of metabolic risk factors among healthy obese adults: 20-year follow-up. *J Am Coll Cardiol.* (2015) 66:871–3. 10.1016/j.jacc.2015.06.014 26271072 PMC4534345

[51] Li Y Schoufour J Wang DD Dhana K Pan A Liu X Healthy lifestyle and life expectancy free of cancer, cardiovascular disease, and type 2 diabetes: prospective cohort study. *BMJ.* (2020) 368:l6669. 10.1136/bmj.l6669 31915124 PMC7190036

[52] O’Doherty MG Cairns K O’Neill V Lamrock F Jørgensen T Brenner H Effect of major lifestyle risk factors, independent and jointly, on life expectancy with and without cardiovascular disease: results from the consortium on health and ageing network of cohorts in Europe and the United States (CHANCES). *Eur J Epidemiol.* (2016) 31:455–68. 10.1007/s10654-015-0112-8 26781655 PMC4901087

[53] Li Y Pan A Wang DD Liu X Dhana K Franco OH Impact of healthy lifestyle factors on life expectancies in the US population. *Circulation.* (2018) 138:345–55. 10.1161/CIRCULATIONAHA 29712712 PMC6207481

