# Supplementary material for: Effects of potential risk factors on the development of cardiometabolic multimorbidity and mortality among the elders in China
Source: Front Cardiovasc Med. 2022 Sep 9;9:966217. doi: 10.3389/fcvm.2022.966217 (PMC9502033; doi:10.3389/fcvm.2022.966217)
Supplement: Supplementary file 1 [file Data_Sheet_1.PDF]

Supplementary Material

STable 1. Characteristics of the subjects by survey wave[a]

Characteristics	2002(n=3436)		2005(n=4264)		2008(n=5194)		2012(n=615)		2014(n=424)	
	n(%)	person-years	n(%)	person-years	n(%)	person-years	n(%)	person-years	n(%)	person-years
**Gender**										
Male	1665(48.5)	14331.59	1726(40.5)	8425.30	2153(41.5)	9777.14	275(44.7)	1090.72	197(46.5)	582.82
Female	1771(51.5)	14692.42	2538(59.5)	10933.24	3041(58.5)	11852.86	340(55.3)	1082.76	227(53.5)	630.21
**Age (y)**										
60-69	845(24.6)	9144.89	616(14.4)	5444.60	576(11.1)	4293.16	86(14.0)	421.45	37(8.7)	128.07
70-79	1412(41.1)	12808.71	323(7.6)	2327.97	321(6.2)	2047.84	105(17.1)	479.01	72(17.0)	249.21
≥80	1179(34.3)	7070.42	3325(78.0)	11585.96	4297(82.7)	15289.01	424(68.9)	1273.02	315(74.3)	835.74
**Marriage**										
In marriage	1958(57.0)	18392.32	1125(26.4)	7669.04	1456(28.0)	8379.05	231(37.6)	1013.41	154(36.3)	495.15
Not in marriage	1478(43.0)	10631.70	3139(73.6)	11689.50	3738(72.0)	13250.95	384(62.4)	1160.07	270(63.7)	717.88
**Residence**										
Rural	2269(66.0)	19585.73	2682(62.9)	12751.71	3605(69.4)	15147.78	548(89.1)	1952.51	314(74.1)	888.36
Town	666(19.4)	5734.81	735(17.2)	3397.22	996(19.2)	4260.52	46(7.5)	148.08	99(23.3)	286.95
City	501(14.6)	3703.47	847(19.9)	3209.60	593(11.4)	2221.70	21(3.4)	72.88	11(2.6)	37.72
**Education**										
No education	1978(57.6)	16062.44	2956(69.3)	12132.67	3560(68.5)	13424.41	406(66.0)	1311.59	278(65.6)	742.30
Elementary school	891(25.9)	7958.91	836(19.6)	4427.88	966(18.6)	4593.93	127(20.7)	491.29	84(19.8)	266.55
Secondary school	454(13.2)	4082.45	373(8.7)	2222.60	574(11.1)	3140.99	74(12.0)	334.07	57(13.4)	186.67
University or above	113(3.3)	920.21	99(2.3)	575.39	94(1.8)	470.68	8(1.3)	36.53	5(1.2)	17.50
**Housing types**										
Purchased	3217(93.6)	27361.67	3971(93.1)	18168.52	4906(94.5)	20491.35	601(97.7)	2122.71	407(96.0)	1162.46
Leased	219(6.4)	1662.34	293(6.9)	1190.02	288(5.5)	1138.65	14(2.3)	50.77	17(4.0)	50.56

**Have one's own bedroom**										
Yes	3063(89.1)	26148.82	3933(92.2)	18181.75	4721(90.9)	19705.04	572(93.0)	2015.09	402(94.8)	1153.87
No	373(10.9)	2875.19	331(7.8)	1176.79	473(9.1)	1924.96	43(7.0)	158.39	22(5.2)	59.15
**Self-assessed economic status**										
Very poor	58(1.7)	466.90	109(2.6)	418.95	191(3.7)	696.14	13(2.1)	57.12	7(1.7)	20.74
Poor	409(11.9)	3234.43	592(13.9)	2542.67	777(15.0)	3296.67	57(9.3)	186.04	31(7.3)	87.51
Fair	2355(68.5)	20193.09	2906(68.2)	13389.26	3514(67.7)	14556.66	447(72.7)	1564.82	310(73.1)	881.45
Rich	576(16.8)	4787.12	611(14.3)	2819.40	665(12.8)	2859.21	92(15.0)	344.71	68(16.0)	200.85
Very rich	38(1.1)	342.48	46(1.1)	188.25	47(0.9)	221.32	6(1.0)	20.78	8(1.9)	22.47
**Pension**										
Yes	650(18.9)	5484.86	629(14.8)	3139.12	531(10.2)	2407.13	53(8.6)	220.86	37(8.7)	118.77
No	2786(81.1)	23539.15	3635(85.2)	16219.42	4663(89.8)	19222.87	562(91.4)	1952.61	387(91.3)	1094.26
**Regular physical activity**										
Yes	1180(34.3)	9971.83	1008(23.6)	5098.17	1154(22.2)	5279.13	76(12.4)	324.37	65(15.3)	199.70
No	2256(65.7)	19052.18	3256(76.4)	14260.37	4040(77.8)	16350.87	539(87.6)	1849.10	359(84.7)	1013.33
**Smoking**										
Non-smoker	2148(62.5)	17992.88	2910(68.2)	12831.45	3606(69.4)	14582.76	458(74.5)	1582.68	325(76.7)	916.40
Smoker	1288(37.5)	11031.13	1354(31.8)	6527.09	1588(30.6)	7047.24	157(25.5)	590.79	99(23.3)	296.63
**Alcohol drinking**										
Non-drinker	2310(67.2)	19645.76	2910(68.2)	12831.45	3696(71.2)	15060.72	470(76.4)	1652.73	332(78.3)	938.27
Drinker	1126(32.8)	9378.25	1354(31.8)	6527.09	1498(28.8)	6569.28	145(23.6)	520.75	92(21.7)	274.76
**Sleep duration**										
≤5.0h	380(11.1)	3237.98	476(11.2)	2289.98	554(10.7)	2287.82	89(14.5)	325.59	33(7.8)	91.44
5.1-7.0h	523(15.2)	4276.70	573(13.4)	2403.25	594(11.4)	2591.31	92(15.0)	348.00	60(14.2)	171.89
7.1-8.0h	1468(42.7)	13246.23	1522(35.7)	8024.54	1831(35.3)	8672.32	275(44.7)	1000.65	207(48.8)	601.48
>8.0h	1065(31.0)	8263.10	1693(39.7)	6640.77	2215(42.6)	8078.56	159(25.9)	498.76	124(29.2)	348.21
**BMI categories**										
Underweight	1271(37.0)	9274.41	1973(46.3)	6987.12	1877(36.1)	6967.74	175(28.5)	518.86	81(19.1)	213.19
Normal	1762(51.3)	15969.80	1969(46.2)	10418.30	2734(52.6)	11763.45	330(53.7)	1208.90	256(60.4)	745.62
Overweight	332(9.7)	3082.11	273(6.4)	1691.84	451(8.7)	2267.00	82(13.3)	342.20	76(17.9)	220.99
Obesity	71(2.1)	697.70	49(1.1)	261.28	132(2.5)	631.81	28(4.6)	103.52	11(2.6)	33.23
**Total**	3436(100)	29024.01	4264(100)	19358.54	5194(100)	21630.00	615(100)	2173.48	424(100)	1213.03

Abbreviations: [a] Data are presented as number (percentage) unless otherwise indicated; BMI, body mass index (calculated as weight in kilograms divided by square of height in meters).

STable 2. Baseline characteristics before and after imputation

Characteristics	Before imputation (n=13933)	After imputation (n=17606)	χ^2	*P* value
**Smoking**				
Non-smoker	9447(67.8)	12103(68.7)	0.548	0.459
Smoker	4486(32.2)	5503(31.3)		
**Alcohol drinking**				
Non-drinker	9718(69.7)	12381(70.3)	0.611	0.434
Drinker	4215(30.3)	5225(29.7)		
**Regular physical activity**				
Yes	3483(25.0)	4228(24.0)	0.926	0.336
No	10450(75.0)	13378(76.0)		
**Sleep duration**				
≤5.0h	1532(11.0)	2015(11.4)	9.075	0.43
5.1-7.0h	3697(25.6)	2332(13.2)		
7.1-8.0h	3448(24.7)	6461(36.7)		
>8.0h	5256(37.7)	6798(38.6)		
**BMI categories**				
Underweight	5377(38.6)	7014(39.8)	11.245	0.259
Normal	7051(50.6)	8742(49.7)		
Overweight	1214(8.7)	1491(8.5)		
Obesity	291(2.1)	359(2.0)		
**Marriage**				
In marriage	4924(35.3)	5509(31.3)	0.064	0.801
Not in marriage	9009(64.7)	12097(68.7)		
**Have one's own bedroom**				
Yes	12691(91.1)	15955(90.6)	1.791	0.181
No	1242(8.9)	1651(9.4)		
**Adequate financial resources**				
Yes	11040(79.2)	13904(79.0)	0.067	0.796
No	2893(20.8)	3702(21.0)		
**Housing types**				
Puechased	13102(94.0)	16538(93.9)	0.155	0.694
Leased	831(6.0)	1068(6.1)		
**Education**				
No education	9178(65.9)	11895(67.6)	15.987	0.067
Elementary school	2904(20.8)	3539(20.1)		
Secondary school	1532(11.0)	1799(10.2)		
University or above	319(2.3)	373(2.1)		

**Pension**				
Yes	1900(13.6)	2269(12.9)	1.261	0.262
No	12033(86.4)	15337(87.1)		
**Self-assessed economic status**				
Very poor	378(2.7)	519(2.9)		
Poor	1866(13.4)	2418(13.7)		
Fair	9532(68.4)	11966(68.0)	7.206	0.969
Rich	2012(14.4)	2511(14.3)		
Very rich	145(1.0)	192(1.1)		

STable 3. Role of factors in transitions between cardiometabolic conditions and mortality after multiple imputation of missing values (n=17606)

**Factors**	**HR (95% CI)**				
	**A(healthy→first disease)**	**B(healthy→mortality)**	**C(first disease→ multimorbidity)**	**D (first disease→mortality)**	**E(multimorbidity→ mortality)**
**Gender**					
Male	1.00 [Ref.]	1.00 [Ref.]	1.00 [Ref.]	1.00 [Ref.]	1.00 [Ref.]
Female	**1.22(1.12-1.34)**	**0.88(0.84-0.93)**	**1.36(1.06-1.73)**	**0.85(0.74-0.98)**	1.37(0.85-2.23)
**Age (y)**					
60-69	1.00 [Ref.]	1.00 [Ref.]	1.00 [Ref.]	1.00 [Ref.]	1.00 [Ref.]
70-79	**1.27(1.16-1.40)**	**1.54(1.40-1.69)**	**0.76(0.60-0.96)**	**2.08(1.75-2.46)**	**2.12(1.32-3.41)**
≥80	0.96(0.87-1.06)	**5.85(5.39-6.34)**	**0.45(0.32-0.62)**	**5.51(4.69-6.48)**	**4.32(2.36-7.92)**
**Marriage**					
In marriage	1.00 [Ref.]	1.00 [Ref.]	1.00 [Ref.]	1.00 [Ref.]	1.00 [Ref.]
Not in marriage	**0.88(0.80-0.96)**	**1.60(1.51-1.68)**	0.97(0.76-1.24)	**1.25(1.10-1.42)**	1.05(0.67-1.65)
**Residence**					
Rural	1.00 [Ref.]	1.00 [Ref.]	1.00 [Ref.]	1.00 [Ref.]	1.00 [Ref.]
Town	**1.20(1.09-1.31)**	0.99(0.94-1.04)	1.13(0.87-1.47)	0.96(0.83-1.11)	1.04(0.64-1.67)
City	**1.28(1.15-1.43)**	**1.12(1.06-1.19)**	**1.47(1.10-1.96)**	0.93(0.77-1.12)	**0.47(0.26-0.88)**
**Regular physical**					
Yes	1.00 [Ref.]	1.00 [Ref.]	1.00 [Ref.]	1.00 [Ref.]	1.00 [Ref.]
No	**1.09(1.01-1.18)**	**1.20(1.15-1.26)**	1.06(0.86-1.31)	1.10(0.97-1.24)	0.74(0.48-1.12)
**Smoking**					
Non-smoker	1.00 [Ref.]	1.00 [Ref.]	1.00 [Ref.]	1.00 [Ref.]	1.00 [Ref.]
Smoker	**1.18(1.07-1.30)**	1.03(0.98-1.09)	1.10(0.84-1.43)	1.04(0.90-1.2)	**2.49(1.43-4.34)**
**Alcohol drinking**					
Non-drinker	1.00 [Ref.]	1.00 [Ref.]	1.00 [Ref.]	1.00 [Ref.]	1.00 [Ref.]
Drinker	0.99(0.91-1.09)	1.00(0.95-1.05)	0.87(0.67-1.13)	1.06(0.93-1.22)	0.94(0.56-1.58)

**Sleep duration**					
≤5.0h	**1.19(1.06-1.33)**	0.99(0.93-1.06)	0.76(0.53-1.08)	**1.23(1.03-1.47)**	0.79(0.37-1.70)
5.1-7.0h	**1.16(1.04-1.29)**	0.96(0.91-1.03)	0.91(0.67-1.23)	1.02(0.85-1.21)	1.20(0.67-2.13)
7.1-8.0h	1.00 [Ref.]	1.00 [Ref.]	1.00 [Ref.]	1.00 [Ref.]	1.00 [Ref.]
>8.0h	1.05(0.96-1.14)	**1.17(1.12-1.22)**	0.92(0.71-1.19)	**1.23(1.08-1.40)**	1.07(0.68-1.70)
**SES**					
Low SES	1.00 [Ref.]	1.00 [Ref.]	1.00 [Ref.]	1.00 [Ref.]	1.00 [Ref.]
Medium SES	**1.14(1.04-1.25)**	**0.95(0.91-0.99)**	0.85(0.64-1.12)	1.04(0.90-1.19)	1.06(0.61-1.83)
High SES	**1.16(1.07-1.26)**	**0.89(0.85-0.93)**	1.07(0.85-1.34)	0.90(0.78-1.03)	1.44(0.91-2.25)
**BMI categories**					
Underweight	**0.87(0.80-0.95)**	**1.20(1.15-1.25)**	**0.73(0.54-0.98)**	**1.21(1.07-1.37)**	1.12(0.65-1.92)
Normal	1.00 [Ref.]	1.00 [Ref.]	1.00 [Ref.]	1.00 [Ref.]	1.00 [Ref.]
Overweight	**1.46(1.31-1.63)**	**0.87(0.81-0.95)**	**1.38(1.06-1.81)**	0.90(0.74-1.10)	1.11(0.66-1.85)
Obesity	**1.29(1.04-1.61)**	1.10(0.95-1.28)	**1.76(1.07-2.90)**	**0.54(0.33-0.88)**	1.44(0.51-4.06)

Abbreviations: SES, socioeconomic status; BMI, body mass index (calculated as weight in kilograms divided by square of height in meters); HR, hazard ratio.

Assessment of socioeconomic status using latent class analysis

In the current study, six economy-related variables reflect different aspects of SES, thus we used these six parameters to generate an overall SES parameter. Latent class analyses with different numbers of latent classes were conducted to select a reasonable model. Log likelihood (Log(L)), Akaike information criterion (AIC), Bayesian information criterion (BIC), adjusted Bayesian information criterion (aBIC), Lo-Mendell-Rubin likelihood ratio test (LMRT), Bootstrap likelihood ratio test (BLRT), and Entropy were used for the model selection. Considering the interpretation of results, we only reported information on models with eight or fewer latent classes. To have a better comparison, comprehensively considering statistics related to model selection, we chose the three-latent-class solution and divided individuals into high, medium, and low SES.

STable 4. Fitting index values in models with different numbers of latent classes

Class	Log(L)	AIC	BIC	aBIC	Entropy	LMRT	BLRT
1	-46264.092	92550.184	92633.146	92598.189			
2	-44223.382	88492.764	88666.230	88593.138	0.718	0.0000	0.0000
**3**	**-42986.136**	**86042.271**	**86306.242**	**86195.015**	**0.827**	**0.0000**	**0.0000**
4	-42919.003	85932.006	86286.481	86137.119	0.825	0.0014	0.0000
5	-42869.303	85856.606	86301.584	86114.088	0.725	0.0000	0.0000
6	-42833.434	85808.868	86344.351	86118.719	0.764	0.1465	0.0000
7	-42817.036	85800.071	86426.059	86162.292	0.693	0.9107	0.0000
8	-42805.377	85800.753	86517.245	86215.343	0.751	0.2815	0.1250

STable 5. Role of factors in transitions between cardiometabolic conditions multimorbidity, and mortality after latent class analysis on economic (n=13933)

Factors	HR (95% CI)				
	A (healthy→first disease)	B (healthy→mortality)	C (first disease→ multimorbidity)	D (first disease→mortality)	E(multimorbidity →mortality)
**Gender**					
Male	1.00 [Ref.]	1.00 [Ref.]	1.00 [Ref.]	1.00 [Ref.]	1.00 [Ref.]
Female	**1.25(1.14-1.38)**	**0.86(0.81-0.90)**	**1.35(1.04-1.77)**	0.86(0.75-1.00)	1.19(0.69-2.06)
**Age (y)**					
60-69	1.00 [Ref.]	1.00 [Ref.]	1.00 [Ref.]	1.00 [Ref.]	1.00 [Ref.]
70-79	**1.28(1.16-1.41)**	**1.44(1.29-1.61)**	**0.74(0.58-0.95)**	**2.32(1.92-2.80)**	**2.28(1.37-3.80)**
≥80	1.06(0.95-1.19)	**6.17(5.62-6.77)**	**0.40(0.28-0.58)**	**6.11(5.12-7.30)**	**4.29(2.28-8.09)**
**Marriage**					
In marriage	1.00 [Ref.]	1.00 [Ref.]	1.00 [Ref.]	1.00 [Ref.]	1.00 [Ref.]
Not in marriage	**0.85(0.78-0.94)**	**1.64(1.55-1.74)**	0.98(0.75-1.29)	**1.24(1.08-1.43)**	1.14(0.69-1.90)
**Residence**					
Rural	1.00 [Ref.]	1.00 [Ref.]	1.00 [Ref.]	1.00 [Ref.]	1.00 [Ref.]
Town	**1.17(1.06-1.29)**	0.99(0.93-1.05)	1.03(0.77-1.37)	1.02(0.88-1.19)	0.98(0.56-1.70)
City	**1.23(1.08-1.39)**	**1.15(1.07-1.23)**	1.29(0.90-1.84)	1.05(0.85-1.29)	0.44(0.19-1.02)
**Class**					
Low	1.00 [Ref.]	1.00 [Ref.]	1.00 [Ref.]	1.00 [Ref.]	1.00 [Ref.]
Medium	**1.27(1.16-1.40)**	**0.93(0.88-0.97)**	1.06(0.81-1.37)	0.93(0.81-1.07)	1.48(0.85-2.56)
High	**1.44(1.26-1.65)**	**0.85(0.78-0.94)**	1.29(0.91-1.83)	**0.68(0.54-0.86)**	1.32(0.61-2.85)
**Regular physical activity**					
Yes	1.00 [Ref.]	1.00 [Ref.]	1.00 [Ref.]	1.00 [Ref.]	1.00 [Ref.]
No	**1.13(1.04-1.23)**	**1.19(1.13-1.25)**	1.10(0.87-1.39)	1.11(0.97-1.27)	0.77(0.47-1.27)
**Smoking**					
Non-smoker	1.00 [Ref.]	1.00 [Ref.]	1.00 [Ref.]	1.00 [Ref.]	1.00 [Ref.]
Smoker	**1.18(1.06-1.30)**	1.03(0.97-1.09)	1.07(0.80-1.43)	1.03(0.88-1.20)	**2.18(1.18-4.04)**

**Alcohol consumption**					
Non-drinker	1.00 [Ref.]	1.00 [Ref.]	1.00 [Ref.]	1.00 [Ref.]	1.00 [Ref.]
Drinker	1.00(0.91-1.11)	1.01(0.95-1.07)	0.87(0.65-1.16)	1.06(0.91-1.23)	1.09(0.61-1.94)
**Sleep duration**					
≤5.0h	**1.14(1.01-1.29)**	0.99(0.92-1.07)	0.86(0.59-1.23)	1.20(0.99-1.46)	0.80(0.37-1.73)
5.1-7.0h	**1.13(1.01-1.27)**	0.95(0.88-1.02)	0.83(0.59-1.17)	1.06(0.88-1.28)	1.12(0.61-2.08)
7.1-8.0h	1.00 [Ref.]	1.00 [Ref.]	1.00 [Ref.]	1.00 [Ref.]	1.00 [Ref.]
>8.0h	1.03(0.94-1.13)	**1.15(1.10-1.21)**	0.86(0.66-1.14)	**1.25(1.09-1.44)**	1.03(0.62-1.70)
**BMI categories**					
Underweight	**0.87(0.79-0.95)**	**1.19(1.14-1.25)**	0.74(0.54-1.02)	**1.20(1.05-1.37)**	1.21(0.67-2.17)
Normal	1.00 [Ref.]	1.00 [Ref.]	1.00 [Ref.]	1.00 [Ref.]	1.00 [Ref.]
Overweight	**1.45(1.29-1.62)**	**0.81(0.74-0.89)**	**1.41(1.07-1.87)**	0.96(0.78-1.18)	1.20(0.68-2.12)
Obesity	**1.32(1.05-1.67)**	1.10(0.92-1.32)	1.67(0.98-2.84)	**0.54(0.32-0.92)**	1.14(0.34-3.82)

Abbreviations: SES, socioeconomic status; BMI, body mass index (calculated as weight in kilograms divided by square of height in meters); HR, hazard ratio.

STable 6. Role of factors in transitions between cardiometabolic conditions and mortality after deleting the disabled population at baseline (n=11207)

Factors	HR (95% CI)				
	A (healthy→first disease)	B (healthy →mortality)	C (first disease→ multimorbidity)	D (first disease→mortality)	E (multimorbidity →mortality)
**Gender**					
Male	1.00 [Ref.]	1.00 [Ref.]	1.00 [Ref.]	1.00 [Ref.]	1.00 [Ref.]
Female	**1.24(1.13-1.37)**	**0.77(0.72-0.82)**	**1.34(1.03-1.74)**	**0.79(0.68-0.93)**	1.19(0.69-2.06)
**Age (y)**					
60-69	1.00 [Ref.]	1.00 [Ref.]	1.00 [Ref.]	1.00 [Ref.]	1.00 [Ref.]
70-79	**1.25(1.13-1.38)**	**1.52(1.35-1.70)**	**0.77(0.60-0.99)**	**2.12(1.76-2.56)**	**2.17(1.31-3.59)**
≥80	**1.13(1.01-1.26)**	**5.89(5.35-6.49)**	**0.43(0.30-0.63)**	**5.33(4.47-6.36)**	**4.53(2.34-8.77)**
**Marriage**					
In marriage	1.00 [Ref.]	1.00 [Ref.]	1.00 [Ref.]	1.00 [Ref.]	1.00 [Ref.]
Not in marriage	**0.87(0.79-0.96)**	**1.61(1.50-1.72)**	0.96(0.73-1.26)	**1.27(1.09-1.47)**	1.15(0.68-1.93)
**Residence**					
Rural	1.00 [Ref.]	1.00 [Ref.]	1.00 [Ref.]	1.00 [Ref.]	1.00 [Ref.]
Town	**1.22(1.11-1.35)**	0.98(0.92-1.05)	1.06(0.80-1.41)	1.02(0.88-1.20)	0.99(0.58-1.67)
City	**1.36(1.20-1.53)**	0.98(0.89-1.07)	**1.42(1.04-1.95)**	0.81(0.64-1.01)	**0.43(0.21-0.89)**
**SES**					
Low SES	1.00 [Ref.]	1.00 [Ref.]	1.00 [Ref.]	1.00 [Ref.]	1.00 [Ref.]
Medium SES	**1.14(1.03-1.27)**	0.97(0.91-1.03)	0.81(0.59-1.11)	1.00(0.85-1.17)	1.21(0.67-2.19)
High SES	**1.15(1.05-1.26)**	**0.83(0.78-0.89)**	1.04(0.82-1.33)	**0.83(0.71-0.96)**	1.23(0.74-2.03)
**Regular physical activity**					
Yes	1.00 [Ref.]	1.00 [Ref.]	1.00 [Ref.]	1.00 [Ref.]	1.00 [Ref.]
No	**1.10(1.01-1.19)**	**1.07(1.01-1.14)**	1.07(0.85-1.35)	1.04(0.91-1.20)	0.72(0.44-1.16)
**Smoking**					
Non-smoker	1.00 [Ref.]	1.00 [Ref.]	1.00 [Ref.]	1.00 [Ref.]	1.00 [Ref.]
Smoker	**1.16(1.05-1.29)**	1.03(0.96-1.10)	1.01(0.76-1.36)	1.00(0.85-1.18)	**2.15(1.15-4.02)**

**Alcohol consumption**					
Non-drinker	1.00 [Ref.]	1.00 [Ref.]	1.00 [Ref.]	1.00 [Ref.]	1.00 [Ref.]
Drinker	1.02(0.92-1.13)	1.00(0.93-1.07)	0.87(0.65-1.17)	1.11(0.95-1.30)	1.15(0.64-2.10)
**Sleep duration**					
≤5.0h	**1.16(1.02-1.31)**	0.95(0.87-1.04)	0.82(0.56-1.20)	1.18(0.96-1.45)	0.85(0.39-1.83)
5.1-7.0h	**1.13(1.01-1.27)**	0.92(0.84-1.00)	0.77(0.54-1.08)	1.05(0.87-1.29)	0.97(0.50-1.89)
7.1-8.0h	1.00 [Ref.]	1.00 [Ref.]	1.00 [Ref.]	1.00 [Ref.]	1.00 [Ref.]
>8.0h	1.05(0.96-1.16)	**1.08(1.02-1.14)**	0.86(0.65-1.13)	**1.21(1.04-1.40)**	1.07(0.64-1.77)
**BMI categories**					
Underweight	**0.85(0.77-0.93)**	**1.21(1.14-1.27)**	0.74(0.53-1.02)	**1.18(1.03-1.36)**	1.24(0.67-2.28)
Normal	1.00 [Ref.]	1.00 [Ref.]	1.00 [Ref.]	1.00 [Ref.]	1.00 [Ref.]
Overweight	**1.49(1.32-1.67)**	**0.81(0.72-0.90)**	**1.41(1.06-1.87)**	0.95(0.76-1.18)	1.25(0.71-2.21)
Obesity	**1.35(1.06-1.72)**	1.11(0.89-1.38)	**1.83(1.05-3.17)**	0.66(0.38-1.14)	1.20(0.36-4.02)

Abbreviations: SES, socioeconomic status; BMI, body mass index (calculated as weight in kilograms divided by square of height in meters); HR, hazard ratio.

STable 7. Association of factors with cardiometabolic conditions and mortality using Cox regression model in female (n=7917)

Factors	First cardiometabolic disease		Cardiometabolic multimorbidity		Mortality	
	n	HR(95%CI)	n	HR(95%CI)	n	HR(95%CI)
**Age (y)**						
60-69	378	1.00 [Ref.]	178	1.00 [Ref.]	186	1.00 [Ref.]
70-79	395	**1.30(1.12-1.50)**	162	**1.30(1.04-1.61)**	413	**2.06(1.73-2.46)**
≥80	654	1.14(0.98-1.34)	165	1.12(0.86-1.45)	4966	**8.99(7.66-10.56)**
**Marriage**						
In marriage	573	1.00 [Ref.]	250	1.00 [Ref.]	592	1.00 [Ref.]
Not in marriage	854	1.00(0.88-1.13)	255	0.89(0.72-1.09)	4973	**1.64(1.49-1.81)**
**Residence**						
Rural	946	1.00 [Ref.]	316	1.00 [Ref.]	3900	1.00 [Ref.]
Town	273	1.07(0.94-1.23)	98	1.08(0.86-1.36)	970	0.98(0.91-1.05)
City	208	**1.33(1.13-1.56)**	91	**1.55(1.21-2.00)**	695	1.08(0.99-1.17)
**SES**						
Low SES	502	1.00 [Ref.]	177	1.00 [Ref.]	2040	1.00 [Ref.]
Medium SES	571	0.94(0.83-1.06)	166	**0.75(0.60-0.93)**	2478	0.98(0.92-1.04)
High SES	354	**0.82(0.71-0.94)**	162	1.01(0.81-1.25)	1047	**0.88(0.82-0.95)**
**Regular physical activity**						
Yes	370	1.00 [Ref.]	134	1.00 [Ref.]	950	1.00 [Ref.]
No	1057	0.95(0.84-1.08)	371	1.16(0.93-1.43)	4615	**1.23(1.15-1.33)**
**Smoking**						
Non-smoker	1264	1.00 [Ref.]	459	1.00 [Ref.]	4923	1.00 [Ref.]
Smoker	163	1.06(0.88-1.28)	46	0.88(0.63-1.23)	642	**1.12(1.02-1.23)**
**Alcohol drinking**						
Non-drinker	1252	1.00 [Ref.]	457	1.00 [Ref.]	4796	1.00 [Ref.]
Drinker	175	0.84(0.71-1.01)	48	**0.69(0.50-0.95)**	769	0.98(0.90-1.07)
**Sleep duration**						
≤5.0h	189	0.97(0.81-1.15)	70	1.01(0.76-1.34)	645	1.00(0.91-1.10)
5.1-7.0h	420	0.87(0.76-1.00)	161	0.92(0.74-1.15)	1293	0.97(0.90-1.05)
7.1-8.0h	405	1.00 [Ref.]	150	1.00 [Ref.]	1184	1.00 [Ref.]
>8.0h	413	0.89(0.78-1.03)	124	0.84(0.66-1.07)	2443	**1.21(1.13-1.30)**
**BMI categories**						
Underweight	462	0.91(0.81-1.03)	110	**0.68(0.54-0.85)**	2845	**1.21(1.14-1.28)**

Normal	747	1.00 [Ref.]	267	1.00 [Ref.]	2329	1.00 [Ref.]
Overweight	170	**1.40(1.19-1.66)**	96	**1.94(1.53-2.46)**	299	**0.85(0.75-0.96)**
Obesity	48	1.27(0.95-1.71)	32	**2.11(1.46-3.06)**	92	0.98(0.79-1.21)

Abbreviations: SES, socioeconomic status; BMI, body mass index (calculated as weight in kilograms divided by square of height in meters); HR, hazard ratio.

STable 8. Association of factors with cardiometabolic conditions and mortality using Cox regression model in male(n=6016)

Factors	First cardiometabolic disease		Cardiometabolic multimorbidity		Mortality	
	n	HR(95%CI)	n	HR(95%CI)	n	HR(95%CI)
**Age (y)**						
60-69	442	1.00 [Ref.]	191	1.00 [Ref.]	314	1.00 [Ref.]
70-79	397	1.09(0.95-1.25)	143	0.97(0.78-1.21)	571	**2.04(1.78-2.34)**
≥80	500	**1.40(1.21-1.62)**	136	1.02(0.79-1.32)	2915	**7.11(6.27-8.07)**
**Marriage**						
In marriage	973	1.00 [Ref.]	335	1.00 [Ref.]	1636	1.00 [Ref.]
Not in marriage	366	0.91(0.80-1.04)	135	**1.27(1.02-1.59)**	2164	**1.58(1.48-1.70)**
**Residence**						
Rural	892	1.00 [Ref.]	304	1.00 [Ref.]	2602	1.00 [Ref.]
Town	271	1.07(0.94-1.23)	85	0.99(0.78-1.27)	711	1.06(0.97-1.15)
City	176	1.09(0.92-1.29)	81	**1.45(1.11-1.88)**	487	**1.18(1.07-1.31)**
**SES**						
Low SES	368	1.00 [Ref.]	113	1.00 [Ref.]	1159	1.00 [Ref.]
Medium SES	246	1.11(0.95-1.31)	78	1.16(0.87-1.55)	873	1.01(0.92-1.10)
High SES	725	1.02(0.89-1.16)	279	1.16(0.93-1.45)	1768	0.95(0.88-1.02)
**Regular physical activity**						
Yes	488	1.00 [Ref.]	169	1.00 [Ref.]	1108	1.00 [Ref.]
No	851	0.94(0.87-1.03)	301	1.13(0.93-1.39)	2692	**1.25(1.17-1.35)**
**Smoking**						
Non-smoker	516	1.00 [Ref.]	165	1.00 [Ref.]	1551	1.00 [Ref.]
Smoker	823	1.03(0.91-1.17)	305	1.04(0.85-1.29)	2249	1.05(0.97-1.13)
**Alcohol drinking**						
Non-drinker	640	1.00 [Ref.]	203	1.00 [Ref.]	1794	1.00 [Ref.]
Drinker	699	0.98(0.87-1.10)	267	1.17(0.95-1.43)	2006	**1.09(1.01-1.17)**
**Sleep duration**						
≤5.0h	130	1.10(0.90-1.35)	38	0.92(0.64-1.32)	378	1.11(0.99-1.26)
5.1-7.0h	412	1.14(0.99-1.31)	160	1.19(0.94-1.49)	950	1.04(0.95-1.14)
7.1-8.0h	377	1.00 [Ref.]	135	1.00 [Ref.]	930	1.00 [Ref.]
>8.0h	420	1.08(0.94-1.24)	137	1.01(0.79-1.29)	1542	**1.19(1.09-1.29)**

**BMI categories**						
Underweight	284	**0.83(0.72-0.95)**	80	**0.73(0.57-0.94)**	1376	**1.24(1.15-1.32)**
Normal	834	1.00 [Ref.]	294	1.00 [Ref.]	2067	1.00 [Ref.]
Overweight	194	**1.28(1.09-1.50)**	80	**1.38(1.07-1.78)**	307	0.89(0.79-1.00)
Obesity	27	1.32(0.90-1.94)	16	**1.98(1.19-3.28)**	50	1.09(0.82-1.45)

Abbreviations: SES, socioeconomic status; BMI, body mass index (calculated as weight in kilograms divided by square of height in meters); HR, hazard ratio.

STable 9. Association of factors with cardiometabolic conditions and mortality using Cox regression model in low SES population (n=4588)

Factors	First cardiometabolic disease		Cardiometabolic multimorbidity		Mortality	
	**n**	**HR(95%CI)**	**n**	**HR(95%CI)**	**n**	**HR(95%CI)**
**Gender**						
Male	368	1.00 [Ref.]	113	1.00 [Ref.]	1159	1.00 [Ref.]
Female	502	1.03(0.87-1.22)	177	1.26(0.94-1.71)	2040	0.92(0.84-1.00)
**Age (y)**						
60-69	250	1.00 [Ref.]	109	1.00 [Ref.]	164	1.00 [Ref.]
70-79	222	1.03(0.86-1.24)	80	0.98(0.73-1.32)	298	**1.89(1.56-2.28)**
≥80	398	**1.24(1.03-1.50)**	101	1.04(0.75-1.44)	2737	**7.28(6.14-8.63)**
**Marriage**						
In marriage	454	1.00 [Ref.]	163	1.00 [Ref.]	754	1.00 [Ref.]
Not in marriage	416	0.96(0.82-1.13)	127	1.01(0.77-1.34)	2445	**1.52(1.38-1.67)**
**Residence**						
Rural	581	1.00 [Ref.]	191	1.00 [Ref.]	2280	1.00 [Ref.]
Town	147	1.06(0.88-1.27)	51	1.05(0.77-1.44)	483	1.01(0.91-1.11)
City	142	**1.47(1.20-1.79)**	48	**1.43(1.01-2.02)**	436	1.03(0.92-1.14)
**Regular physical activity**						
Yes	240	1.00 [Ref.]	72	1.00 [Ref.]	603	1.00 [Ref.]
No	630	0.94(0.80-1.10)	217	1.24(0.93-1.65)	2595	**1.32(1.21-1.45)**
**Smoking**						
Non-smoker	588	1.00 [Ref.]	193	1.00 [Ref.]	2295	1.00 [Ref.]
Smoker	282	0.94(0.78-1.13)	97	1.08(0.78-1.49)	904	1.05(0.95-1.17)
**Alcohol drinking**						
Non-drinker	631	1.00 [Ref.]	212	1.00 [Ref.]	2348	1.00 [Ref.]
Drinker	239	0.87(0.73-1.05)	78	0.87(0.64-1.20)	851	1.02(0.93-1.12)
**Sleep duration**						
≤5.0h	139	1.14(0.92-1.41)	50	1.20(0.84-1.73)	433	1.05(0.93-1.18)

5.1-7.0h	266	0.99(0.83-1.19)	101	1.09(0.80-1.48)	825	1.00(0.90-1.11)
7.1-8.0h	212	1.00 [Ref.]	72	1.00 [Ref.]	662	1.00 [Ref.]
>8.0h	253	1.11(0.92-1.34)	67	0.92(0.65-1.28)	1279	**1.24(1.13-1.37)**
**BMI categories**						
Underweight	263	**0.80(0.69-0.93)**	58	**0.54(0.40-0.73)**	1565	**1.18(1.09-1.27)**
Normal	492	1.00 [Ref.]	174	1.00 [Ref.]	1413	1.00 [Ref.]
Overweight	94	1.23(0.98-1.54)	47	**1.54(1.11-2.14)**	184	0.94(0.81-1.10)
Obesity	21	1.50(0.96-2.33)	11	**1.88(1.01-3.48)**	37	1.03(0.74-1.42)

Abbreviations: SES, socioeconomic status; BMI, body mass index (calculated as weight in kilograms divided by square of height in meters); HR, hazard ratio.

STable 10. Association of factors with cardiometabolic conditions and mortality using Cox regression model in medium SES population (n=4548)

Factors	First cardiometabolic disease		Cardiometabolic multimorbidity		Mortality	
	**n**	**HR(95%CI)**	**n**	**HR(95%CI)**	**n**	**HR(95%CI)**
**Gender**						
Male	246	1.00 [Ref.]	78	1.00 [Ref.]	873	1.00 [Ref.]
Female	571	0.96(0.80-1.15)	166	0.85(0.61-1.19)	2478	**0.90(0.82-0.98)**
**Age (y)**						
60-69	179	1.00 [Ref.]	74	1.00 [Ref.]	94	1.00 [Ref.]
70-79	225	**1.32(1.08-1.62)**	81	1.20(0.87-1.66)	267	**2.27(1.79-2.88)**
≥80	413	1.19(0.97-1.48)	89	0.99(0.68-1.43)	2990	**9.26(7.44-11.52)**
**Marriage**						
In marriage	355	1.00 [Ref.]	131	1.00 [Ref.]	526	1.00 [Ref.]
Not in marriage	462	1.07(0.90-1.27)	113	0.97(0.72-1.31)	2825	**1.63(1.47-1.81)**
**Residence**						
Rural	596	1.00 [Ref.]	186	1.00 [Ref.]	2481	1.00 [Ref.]
Town	160	1.15(0.96-1.37)	38	0.84(0.59-1.20)	613	1.04(0.95-1.13)
City	61	1.09(0.83-1.43)	20	1.07(0.66-1.72)	257	1.10(0.97-1.25)
**Regular physical activity**						
Yes	155	1.00 [Ref.]	43	1.00 [Ref.]	560	1.00 [Ref.]
No	662	1.19(0.99-1.42)	201	1.32(0.94-1.86)	2791	**1.15(1.04-1.26)**
**Smoking**						
Non-smoker	604	1.00 [Ref.]	185	1.00 [Ref.]	2574	1.00 [Ref.]
Smoker	213	**1.24(1.00-1.54)**	59	1.03(0.70-1.54)	777	1.07(0.96-1.19)
**Alcohol drinking**						
Non-drinker	626	1.00 [Ref.]	184	1.00 [Ref.]	2575	1.00 [Ref.]
Drinker	191	0.93(0.76-1.14)	60	1.05(0.73-1.50)	776	1.00(0.90-1.10)
**Sleep duration**						
≤5.0h	90	0.98(0.76-1.25)	22	0.78(0.48-1.26)	341	0.99(0.87-1.12)
5.1-7.0h	226	0.97(0.81-1.17)	78	1.05(0.76-1.45)	746	0.98(0.89-1.09)
7.1-8.0h	226	1.00 [Ref.]	72	1.00 [Ref.]	752	1.00 [Ref.]
>8.0h	275	0.94(0.78-1.12)	72	0.84(0.60-1.17)	1512	**1.15(1.05-1.25)**
**BMI categories**						
Underweight	257	0.99(0.85-1.16)	55	0.76(0.55-1.04)	1534	**1.14(1.06-1.22)**

Normal	437	1.00 [Ref.]	133	1.00 [Ref.]	1557	1.00 [Ref.]
Overweight	98	**1.43(1.14-1.78)**	42	**1.84(1.29-2.62)**	204	**0.79(0.68-0.92)**
Obesity	25	1.19(0.79-1.78)	14	**1.84(1.05-3.21)**	56	0.91(0.70-1.19)

Abbreviations: SES, socioeconomic status; BMI, body mass index (calculated as weight in kilograms divided by square of height in meters); HR, hazard ratio.

STable 11. Association of factors with cardiometabolic conditions and mortality using Cox regression model in high SES population (n=4797)

Factors	First cardiometabolic disease		Cardiometabolic multimorbidity		Mortality	
	**n**	**HR(95%CI)**	**n**	**HR(95%CI)**	**n**	**HR(95%CI)**
**Gender**						
Male	725	1.00 [Ref.]	279	1.00 [Ref.]	1768	1.00 [Ref.]
Female	354	0.98(0.84-1.14)	162	1.11(0.88-1.41)	1047	**0.86(0.78-0.94)**
**Age (y)**						
60-69	391	1.00 [Ref.]	186	1.00 [Ref.]	242	1.00 [Ref.]
70-79	345	**1.24(1.07-1.44)**	144	1.10(0.88-1.38)	419	**2.08(1.77-2.43)**
≥80	343	**1.31(1.10-1.55)**	111	1.05(0.79-1.38)	2154	**7.89(6.80-9.16)**
**Marriage**						
In marriage	737	1.00 [Ref.]	291	1.00 [Ref.]	948	1.00 [Ref.]
Not in marriage	342	**0.85(0.73-0.99)**	150	1.11(0.88-1.39)	1867	**1.65(1.51-1.81)**
**Residence**						
Rural	661	1.00 [Ref.]	243	1.00 [Ref.]	1741	1.00 [Ref.]
Town	237	1.01(0.87-1.17)	94	1.13(0.89-1.44)	585	1.00(0.91-1.10)
City	181	1.04(0.88-1.24)	104	**1.67(1.31-2.14)**	489	**1.26(1.13-1.40)**
**Regular physical activity**						
Yes	463	1.00 [Ref.]	188	1.00 [Ref.]	895	1.00 [Ref.]
No	616	**0.85(0.75-0.97)**	253	1.04(0.85-1.28)	1920	**1.26(1.16-1.37)**
**Smoking**						
Non-smoker	588	1.00 [Ref.]	246	1.00 [Ref.]	1605	1.00 [Ref.]
Smoker	491	1.00(0.86-1.17)	195	0.95(0.75-1.20)	1210	**1.11(1.01-1.23)**
**Alcohol drinking**						
Non-drinker	635	1.00 [Ref.]	264	1.00 [Ref.]	1667	1.00 [Ref.]
Drinker	444	0.99(0.85-1.15)	177	1.09(0.87-1.38)	1148	**1.11(1.01-1.22)**
**Sleep duration**						
≤5.0h	90	1.00(0.79-1.26)	36	0.94(0.65-1.36)	249	1.11(0.96-1.28)
5.1-7.0h	340	1.04(0.89-1.21)	142	1.02(0.81-1.29)	672	1.01(0.91-1.13)
7.1-8.0h	344	1.00 [Ref.]	141	1.00 [Ref.]	700	1.00 [Ref.]
>8.0h	305	0.93(0.80-1.09)	122	0.97(0.76-1.24)	1194	**1.22(1.11-1.35)**
**BMI categories**						

Underweight	226	**0.82(0.71-0.96)**	77	0.82(0.63-1.06)	1122	**1.38(1.27-1.50)**
Normal	652	1.00 [Ref.]	254	1.00 [Ref.]	1426	1.00 [Ref.]
Overweight	172	**1.33(1.12-1.57)**	87	**1.60(1.25-2.04)**	218	0.88(0.76-1.02)
Obesity	29	1.28(0.88-1.86)	23	**2.32(1.51-3.58)**	49	1.14(0.85-1.51)

Abbreviations: SES, socioeconomic status; BMI, body mass index (calculated as weight in kilograms divided by square of height in meters); HR, hazard ratio.

STable 12. Role of factors in transitions between cardiometabolic conditions and mortality using multi-state model in female (n=7917)

Factors	HR (95% CI)				
	**A (healthy→first disease)**	**B (healthy→ mortality)**	**C (first disease→ multimorbidity)**	**D (first disease→ mortality)**	**E (multimorbidity→ mortality)**
**Age (y)**					
60-69	1.00 [Ref.]	1.00 [Ref.]	1.00 [Ref.]	1.00 [Ref.]	1.00 [Ref.]
70-79	**1.52(1.31-1.77)**	1.17(0.99-1.39)	0.89(0.62-1.27)	**2.02(1.54-2.66)**	1.68(0.79-3.61)
≥80	0.98(0.83-1.15)	**6.00(5.22-6.88)**	**0.48(0.29-0.79)**	**6.03(4.62-7.87)**	**3.50(1.26-9.74)**
**Marriage**					
In marriage	1.00 [Ref.]	1.00 [Ref.]	1.00 [Ref.]	1.00 [Ref.]	1.00 [Ref.]
Not in marriage	0.99(0.86-1.13)	**1.54(1.40-1.71)**	0.85(0.61-1.20)	**1.24(1.01-1.52)**	1.24(0.61-2.54)
**Residence**					
Rural	1.00 [Ref.]	1.00 [Ref.]	1.00 [Ref.]	1.00 [Ref.]	1.00 [Ref.]
Town	**1.26(1.10-1.45)**	0.96(0.89-1.04)	1.09(0.74-1.60)	0.91(0.74-1.13)	1.25(0.61-2.60)
City	**1.59(1.36-1.86)**	1.04(0.96-1.14)	**1.64(1.09-2.45)**	0.95(0.73-1.25)	0.85(0.34-2.15)
**SES**					
Low SES	1.00 [Ref.]	1.00 [Ref.]	1.00 [Ref.]	1.00 [Ref.]	1.00 [Ref.]
Medium SES	**1.15(1.02-1.30)**	**0.92(0.87-0.98)**	0.80(0.56-1.16)	0.98(0.81-1.18)	1.34(0.65-2.77)
High SES	1.11(0.97-1.27)	**0.82(0.76-0.89)**	1.34(0.95-1.87)	0.82(0.65-1.04)	1.10(0.53-2.27)
**Regular physical activity**					
Yes	1.00 [Ref.]	1.00 [Ref.]	1.00 [Ref.]	1.00 [Ref.]	1.00 [Ref.]
No	**1.30(1.15-1.48)**	**1.13(1.05-1.22)**	**1.43(1.05-1.95)**	1.06(0.87-1.29)	1.15(0.60-2.20)
**Smoking**					
Non-smoker	1.00 [Ref.]	1.00 [Ref.]	1.00 [Ref.]	1.00 [Ref.]	1.00 [Ref.]
Smoker	1.15(0.96-1.38)	1.09(0.99-1.20)	1.24(0.71-2.16)	1.12(0.83-1.49)	1.86(0.65-5.31)
**Alcohol drinking**					
Non-drinker	1.00 [Ref.]	1.00 [Ref.]	1.00 [Ref.]	1.00 [Ref.]	1.00 [Ref.]
Drinker	0.91(0.76-1.08)	0.97(0.89-1.06)	0.52(0.27-1.00)	0.94(0.72-1.23)	1.33(0.40-4.44)
**Sleep duration**					

≤5.0h	1.15(0.98-1.36)	0.96(0.87-1.06)	0.89(0.56-1.41)	1.15(0.88-1.49)	0.98(0.36-2.70)
5.1-7.0h	1.06(0.90-1.24)	0.95(0.86-1.04)	0.88(0.55-1.41)	1.18(0.91-1.55)	1.06(0.42-2.68)
7.1-8.0h	1.00 [Ref.]	1.00 [Ref.]	1.00 [Ref.]	1.00 [Ref.]	1.00 [Ref.]
>8.0h	1.00(0.87-1.13)	**1.18(1.11-1.26)**	0.84(0.56-1.26)	1.20(0.99-1.46)	0.99(0.45-2.17)
**BMI categories**					
Underweight	0.93(0.83-1.05)	**1.18(1.11-1.25)**	0.79(0.52-1.20)	**1.19(1.00-1.42)**	1.42(0.61-3.31)
Normal	1.00 [Ref.]	1.00 [Ref.]	1.00 [Ref.]	1.00 [Ref.]	1.00 [Ref.]
Overweight	**1.57(1.33-1.86)**	**0.82(0.72-0.94)**	**1.70(1.15-2.53)**	0.77(0.56-1.06)	1.32(0.59-2.96)
Obesity	**1.41(1.05-1.89)**	1.04(0.84-1.30)	**2.84(1.57-5.15)**	**0.46(0.23-0.93)**	0.97(0.22-4.32)

Abbreviations: SES, socioeconomic status; BMI, body mass index (calculated as weight in kilograms divided by square of height in meters); HR, hazard ratio.

STable 13. Role of factors in transitions between cardiometabolic conditions and mortality using multi-state model in male (n=6016)

**Factors**	**HR (95% CI)**				
	**A (healthy→first disease)**	**B (healthy→ mortality)**	**C (first disease→ multimorbidity)**	**D (first disease→ mortality)**	**E (multimorbidity→ mortality)**
**Age (y)**					
60-69	1.00 [Ref.]	1.00 [Ref.]	1.00 [Ref.]	1.00 [Ref.]	1.00 [Ref.]
70-79	**1.19(1.04-1.36)**	**1.50(1.30-1.73)**	0.71(0.49-1.02)	**2.17(1.70-2.76)**	**3.45(1.71-6.95)**
≥80	**1.22(1.06-1.41)**	**5.64(5.00-6.35)**	**0.37(0.21-0.64)**	**5.16(4.12-6.46)**	**8.39(2.84-24.77)**
**Marriage**					
In marriage	1.00 [Ref.]	1.00 [Ref.]	1.00 [Ref.]	1.00 [Ref.]	1.00 [Ref.]
Not in marriage	**0.83(0.73-0.95)**	**1.65(1.53-1.78)**	1.28(0.83-1.96)	1.20(0.99-1.45)	1.41(0.63-3.12)
**Residence**					
Rural	1.00 [Ref.]	1.00 [Ref.]	1.00 [Ref.]	1.00 [Ref.]	1.00 [Ref.]
Town	**1.19(1.04-1.36)**	1.02(0.93-1.12)	1.11(0.74-1.67)	1.05(0.85-1.30)	0.79(0.35-1.76)
City	**1.19(1.01-1.41)**	**1.22(1.10-1.36)**	1.33(0.83-2.14)	0.88(0.66-1.17)	**0.23(0.08-0.67)**
**SES**					
Low SES	1.00 [Ref.]	1.00 [Ref.]	1.00 [Ref.]	1.00 [Ref.]	1.00 [Ref.]
Medium SES	**1.33(1.13-1.56)**	0.94(0.86-1.03)	0.90(0.51-1.57)	1.14(0.90-1.45)	0.92(0.32-2.69)
High SES	**1.28(1.13-1.45)**	**0.88(0.82-0.96)**	1.00(0.71-1.42)	0.88(0.73-1.07)	1.24(0.61-2.51)
**Regular physical activity**					
Yes	1.00 [Ref.]	1.00 [Ref.]	1.00 [Ref.]	1.00 [Ref.]	1.00 [Ref.]
No	1.11(0.99-1.25)	**1.20(1.11-1.29)**	0.99(0.72-1.37)	1.13(0.95-1.35)	0.57(0.26-1.26)
**Smoking**					
Non-smoker	1.00 [Ref.]	1.00 [Ref.]	1.00 [Ref.]	1.00 [Ref.]	1.00 [Ref.]
Smoker	**1.13(1.00-1.28)**	1.00(0.93-1.09)	1.08(0.75-1.55)	0.94(0.78-1.14)	2.44(0.99-6.01)
**Alcohol drinking**					
Non-drinker	1.00 [Ref.]	1.00 [Ref.]	1.00 [Ref.]	1.00 [Ref.]	1.00 [Ref.]
Drinker	1.02(0.90-1.15)	1.06(0.97-1.14)	0.99(0.70-1.39)	1.13(0.94-1.36)	0.98(0.45-2.15)
**Sleep duration**					

≤5.0h	1.20(0.99-1.45)	1.02(0.90-1.15)	0.86(0.47-1.58)	1.19(0.89-1.58)	0.69(0.21-2.32)
5.1-7.0h	**1.28(1.09-1.50)**	0.93(0.83-1.04)	0.78(0.47-1.29)	0.96(0.73-1.25)	1.38(0.53-3.60)
7.1-8.0h	1.00 [Ref.]	1.00 [Ref.]	1.00 [Ref.]	1.00 [Ref.]	1.00 [Ref.]
>8.0h	1.12(0.98-1.27)	**1.10(1.01-1.18)**	0.97(0.66-1.43)	**1.29(1.06-1.57)**	1.17(0.52-2.63)
**BMI categories**					
Underweight	**0.85(0.74-0.97)**	**1.19(1.11-1.28)**	0.80(0.49-1.31)	1.16(0.95-1.42)	1.00(0.41-2.42)
Normal	1.00 [Ref.]	1.00 [Ref.]	1.00 [Ref.]	1.00 [Ref.]	1.00 [Ref.]
Overweight	**1.46(1.25-1.71)**	**0.78(0.68-0.89)**	1.34(0.89-2.01)	1.05(0.80-1.39)	1.25(0.56-2.80)
Obesity	1.40(0.96-2.06)	1.17(0.87-1.59)	0.58(0.14-2.38)	0.57(0.25-1.28)	2.46(0.28-21.42)

Abbreviations: SES, socioeconomic status; BMI, body mass index (calculated as weight in kilograms divided by square of height in meters); HR, hazard ratio.

STable 14. Role of factors in transitions between cardiometabolic conditions and mortality using multi-state model in low SES population (n=4588)

**Factors**	**HR (95% CI)**				
	**A (healthy→first disease)**	**B (healthy→mortality)**	**C (first disease→ multimorbidity)**	**D (first disease→ mortality)**	**E (multimorbidity→ mortality)**
**Gender**					
Male	1.00 [Ref.]	1.00 [Ref.]	1.00 [Ref.]	1.00 [Ref.]	1.00 [Ref.]
Female	**1.34(1.13-1.58)**	**0.87(0.79-0.95)**	1.44(0.90-2.30)	0.93(0.72-1.21)	0.84(0.29-2.42)
**Age (y)**					
60-69	1.00 [Ref.]	1.00 [Ref.]	1.00 [Ref.]	1.00 [Ref.]	1.00 [Ref.]
70-79	1.15(0.95-1.38)	**1.28(1.05-1.55)**	0.89(0.55-1.45)	**2.39(1.69-3.37)**	**3.10(1.10-8.73)**
≥80	1.02(0.84-1.23)	**5.59(4.78-6.54)**	**0.33(0.17-0.65)**	**6.32(4.68-8.54)**	2.76(0.63-12.02)
**Marriage**					
In marriage	1.00 [Ref.]	1.00 [Ref.]	1.00 [Ref.]	1.00 [Ref.]	1.00 [Ref.]
Not in marriage	0.84(0.71-1.00)	**1.61(1.46-1.79)**	0.95(0.58-1.55)	0.96(0.75-1.25)	1.95(0.63-6.05)
**Residence**					
Rural	1.00 [Ref.]	1.00 [Ref.]	1.00 [Ref.]	1.00 [Ref.]	1.00 [Ref.]
Town	**1.20(1.00-1.44)**	0.99(0.89-1.10)	1.27(0.77-2.12)	0.88(0.65-1.19)	1.16(0.37-3.61)
City	**1.57(1.29-1.91)**	1.02(0.91-1.14)	1.51(0.84-2.69)	0.98(0.70-1.37)	0.27(0.05-1.56)
**Regular physical activity**					
Yes	1.00 [Ref.]	1.00 [Ref.]	1.00 [Ref.]	1.00 [Ref.]	1.00 [Ref.]
No	1.14(0.97-1.33)	**1.24(1.13-1.36)**	1.09(0.72-1.63)	**1.36(1.08-1.71)**	0.45(0.18-1.12)
**Smoking**					
Non-smoker	1.00 [Ref.]	1.00 [Ref.]	1.00 [Ref.]	1.00 [Ref.]	1.00 [Ref.]
Smoker	1.13(0.94-1.35)	1.00(0.90-1.12)	1.07(0.63-1.83)	0.95(0.73-1.25)	2.52(0.92-6.87)
**Alcohol drinking**					
Non-drinker	1.00 [Ref.]	1.00 [Ref.]	1.00 [Ref.]	1.00 [Ref.]	1.00 [Ref.]
Drinker	0.96(0.80-1.15)	0.99(0.89-1.10)	0.88(0.50-1.55)	0.95(0.73-1.23)	0.60(0.18-2.05)
**Sleep duration**					
≤5.0h	**1.43(1.15-1.77)**	1.00(0.88-1.14)	0.88(0.48-1.63)	0.97(0.69-1.36)	0.40(0.07-2.13)

5.1-7.0h	**1.28(1.07-1.54)**	0.95(0.85-1.06)	0.90(0.55-1.50)	0.91(0.68-1.21)	1.78(0.71-4.44)
7.1-8.0h	1.00 [Ref.]	1.00 [Ref.]	1.00 [Ref.]	1.00 [Ref.]	1.00 [Ref.]
>8.0h	**1.32(1.10-1.60)**	**1.17(1.06-1.29)**	0.78(0.45-1.35)	1.19(0.90-1.56)	2.67(0.95-7.49)
**BMI categories**					
Underweight	**0.80(0.69-0.94)**	**1.15(1.06-1.24)**	**0.48(0.25-0.90)**	1.14(0.91-1.44)	**4.76(1.17-19.46)**
Normal	1.00 [Ref.]	1.00 [Ref.]	1.00 [Ref.]	1.00 [Ref.]	1.00 [Ref.]
Overweight	**1.32(1.05-1.65)**	0.89(0.75-1.05)	1.55(0.91-2.64)	0.88(0.59-1.32)	2.10(0.71-6.24)
Obesity	**1.62(1.04-2.52)**	1.16(0.81-1.65)	**3.08(1.27-7.45)**	0.58(0.24-1.42)	-

Abbreviations: SES, socioeconomic status; BMI, body mass index (calculated as weight in kilograms divided by square of height in meters); HR, hazard ratio; -, results cannot be fitted due to the small sample size.

STable 15. Role of factors in transitions between cardiometabolic conditions and mortality using multi-state model in medium SES population (n=4548)

**Factors**	**HR (95% CI)**				
	**A (healthy→first disease)**	**B (healthy→ mortality)**	**C (first disease→ multimorbidity)**	**D (first disease→ mortality)**	**E (multimorbidity→ mortality)**
**Gender**					
Male	1.00 [Ref.]	1.00 [Ref.]	1.00 [Ref.]	1.00 [Ref.]	1.00 [Ref.]
Female	**1.20(1.01-1.43)**	**0.86(0.78-0.95)**	1.48(0.86-2.53)	0.82(0.63-1.08)	0.69(0.20-2.37)
**Age (y)**					
60-69	1.00 [Ref.]	1.00 [Ref.]	1.00 [Ref.]	1.00 [Ref.]	1.00 [Ref.]
70-79	**1.36(1.11-1.66)**	**1.56(1.25-1.96)**	0.69(0.39-1.21)	**2.31(1.59-3.34)**	**12.80(2.83-57.90)**
≥80	0.96(0.78-1.19)	**7.13(5.88-8.64)**	0.51(0.25-1.04)	**6.64(4.67-9.46)**	**19.78(3.37-116.01)**
**Marriage**					
In marriage	1.00 [Ref.]	1.00 [Ref.]	1.00 [Ref.]	1.00 [Ref.]	1.00 [Ref.]
Not in marriage	0.95(0.80-1.14)	**1.63(1.46-1.84)**	0.69(0.40-1.19)	**1.43(1.11-1.82)**	1.09(0.33-3.62)
**Residence**					
Rural	1.00 [Ref.]	1.00 [Ref.]	1.00 [Ref.]	1.00 [Ref.]	1.00 [Ref.]
Town	**1.26(1.05-1.50)**	1.01(0.92-1.11)	0.95(0.53-1.69)	1.03(0.80-1.33)	0.91(0.24-3.46)
City	1.19(0.91-1.55)	1.12(0.98-1.28)	0.87(0.38-2.01)	0.89(0.59-1.34)	-
**Regular physical activity**					
Yes	1.00 [Ref.]	1.00 [Ref.]	1.00 [Ref.]	1.00 [Ref.]	1.00 [Ref.]
No	**1.42(1.20-1.69)**	**1.11(1.01-1.22)**	0.90(0.53-1.50)	1.09(0.83-1.44)	1.06(0.40-2.78)
**Smoking**					
Non-smoker	1.00 [Ref.]	1.00 [Ref.]	1.00 [Ref.]	1.00 [Ref.]	1.00 [Ref.]
Smoker	**1.44(1.16-1.77)**	1.00(0.89-1.12)	**2.02(1.01-4.06)**	1.20(0.88-1.63)	0.77(0.11-5.29)
**Alcohol drinking**					
Non-drinker	1.00 [Ref.]	1.00 [Ref.]	1.00 [Ref.]	1.00 [Ref.]	1.00 [Ref.]
Drinker	0.94(0.77-1.15)	1.01(0.90-1.12)	0.66(0.33-1.28)	0.86(0.64-1.16)	1.42(0.33-6.21)
**Sleep duration**					
≤5.0h	1.08(0.85-1.39)	0.93(0.81-1.06)	0.64(0.26-1.58)	1.25(0.86-1.82)	0.22(0.02-2.26)

5.1-7.0h	1.09(0.91-1.32)	0.93(0.84-1.03)	1.25(0.71-2.19)	1.06(0.80-1.42)	0.54(0.18-1.66)
7.1-8.0h	1.00 [Ref.]	1.00 [Ref.]	1.00 [Ref.]	1.00 [Ref.]	1.00 [Ref.]
>8.0h	1.01(0.84-1.21)	**1.09(1.00-1.20)**	0.93(0.49-1.76)	1.25(0.96-1.61)	0.44(0.11-1.78)
**BMI categories**					
Underweight	0.95(0.81-1.12)	**1.12(1.04-1.21)**	0.71(0.37-1.35)	1.09(0.87-1.36)	1.99(0.34-11.85)
Normal	1.00 [Ref.]	1.00 [Ref.]	1.00 [Ref.]	1.00 [Ref.]	1.00 [Ref.]
Overweight	**1.55(1.24-1.93)**	**0.74(0.63-0.87)**	1.75(0.96-3.16)	0.97(0.67-1.41)	**5.27(1.34-20.75)**
Obesity	1.27(0.84-1.90)	0.95(0.71-1.26)	1.98(0.77-5.14)	0.55(0.24-1.26)	4.21(0.40-44.51)

Abbreviations: SES, socioeconomic status; BMI, body mass index (calculated as weight in kilograms divided by square of height in meters); HR, hazard ratio; -, results cannot be fitted due to the small sample size.

STable 16. Role of factors in transitions between cardiometabolic conditions and mortality using multi-state model in high SES population (n=4797)

**Factors**	**HR (95% CI)**				
	**A (healthy→ first disease)**	**B (healthy→mortality)**	**C (first disease → multimorbidity)**	**D (first disease →mortality)**	**E (multimorbidity →mortality)**
**Gender**					
Male	1.00 [Ref.]	1.00 [Ref.]	1.00 [Ref.]	1.00 [Ref.]	1.00 [Ref.]
Female	**1.29(1.11-1.50)**	**0.79(0.72-0.87)**	1.37(0.94-2.01)	0.83(0.64-1.07)	1.76(0.68-4.57)
**Age (y)**					
60-69	1.00 [Ref.]	1.00 [Ref.]	1.00 [Ref.]	1.00 [Ref.]	1.00 [Ref.]
70-79	**1.48(1.28-1.71)**	**1.38(1.17-1.61)**	0.75(0.52-1.07)	**2.07(1.59-2.70)**	**2.15(1.04-4.47)**
≥80	**1.27(1.07-1.50)**	**5.56(4.85-6.36)**	**0.41(0.23-0.73)**	**5.16(3.97-6.71)**	**5.37(1.88-15.33)**
**Marriage**					
In marriage	1.00 [Ref.]	1.00 [Ref.]	1.00 [Ref.]	1.00 [Ref.]	1.00 [Ref.]
Not in marriage	**0.79(0.68-0.93)**	**1.66(1.51-1.84)**	1.28(0.86-1.91)	**1.36(1.08-1.72)**	0.79(0.36-1.73)
**Residence**					
Rural	1.00 [Ref.]	1.00 [Ref.]	1.00 [Ref.]	1.00 [Ref.]	1.00 [Ref.]
Town	**1.20(1.04-1.40)**	0.96(0.86-1.06)	1.00(0.65-1.52)	1.02(0.80-1.30)	0.90(0.33-2.47)
City	**1.29(1.08-1.52)**	**1.25(1.12-1.40)**	1.49(0.98-2.27)	0.92(0.67-1.26)	0.87(0.36-2.11)
**Regular physical activity**					
Yes	1.00 [Ref.]	1.00 [Ref.]	1.00 [Ref.]	1.00 [Ref.]	1.00 [Ref.]
No	1.05(0.93-1.19)	**1.18(1.09-1.29)**	1.21(0.87-1.67)	0.98(0.80-1.20)	0.67(0.29-1.52)
**Smoking**					
Non-smoker	1.00 [Ref.]	1.00 [Ref.]	1.00 [Ref.]	1.00 [Ref.]	1.00 [Ref.]
Smoker	**1.19(1.03-1.39)**	1.04(0.94-1.15)	0.89(0.60-1.33)	0.92(0.71-1.18)	**4.72(1.46-15.31)**
**Alcohol drinking**					
Non-drinker	1.00 [Ref.]	1.00 [Ref.]	1.00 [Ref.]	1.00 [Ref.]	1.00 [Ref.]
Drinker	1.13(0.98-1.32)	1.02(0.93-1.13)	0.92(0.62-1.36)	**1.37(1.06-1.76)**	0.85(0.32-2.28)
**Sleep duration**					
≤5.0h	1.18(0.93-1.49)	0.98(0.84-1.14)	0.89(0.49-1.63)	1.19(0.83-1.71)	1.66(0.56-4.94)

5.1-7.0h	**1.32(1.13-1.53)**	0.92(0.83-1.03)	0.99(0.68-1.44)	0.87(0.67-1.13)	1.27(0.57-2.83)
7.1-8.0h	1.00 [Ref.]	1.00 [Ref.]	1.00 [Ref.]	1.00 [Ref.]	1.00 [Ref.]
>8.0h	1.12(0.96-1.32)	**1.13(1.02-1.25)**	0.94(0.62-1.43)	1.15(0.90-1.47)	0.77(0.28-2.11)
**BMI categories**					
Underweight	**0.85(0.73-0.99)**	**1.34(1.23-1.45)**	1.04(0.66-1.64)	**1.39(1.10-1.75)**	0.84(0.37-1.92)
Normal	1.00 [Ref.]	1.00 [Ref.]	1.00 [Ref.]	1.00 [Ref.]	1.00 [Ref.]
Overweight	**1.60(1.35-1.90)**	**0.81(0.69-0.95)**	1.23(0.82-1.86)	0.91(0.65-1.27)	0.64(0.24-1.75)
Obesity	1.43(0.99-2.08)	1.25(0.92-1.68)	1.10(0.40-3.04)	0.41(0.13-1.28)	1.21(0.23-6.34)

Abbreviations: SES, socioeconomic status; BMI, body mass index (calculated as weight in kilograms divided by square of height in meters); HR, hazard [illegible]

STable 17. Role of factors in transitions between cardiometabolic conditions and mortality after excluding cardiometabolic disease occurring within two years after recruitment (n=12398)

**Factors**	**HR (95% CI)**				
	**A (healthy→ first disease)**	**B (healthy→mortality)**	**C (first disease → multimorbidity)**	**D (first disease →mortality)**	**E (multimorbidity →mortality)**
**Gender**					
Male	1.00 [Ref.]	1.00 [Ref.]	1.00 [Ref.]	1.00 [Ref.]	1.00 [Ref.]
Female	**1.23(1.12-1.36)**	**0.85(0.80-0.89)**	**1.41(1.09-1.84)**	**0.84(0.73-0.98)**	1.25(0.73-2.14)
**Age (y)**					
60-69	1.00 [Ref.]	1.00 [Ref.]	1.00 [Ref.]	1.00 [Ref.]	1.00 [Ref.]
70-79	**1.27(1.15-1.40)**	**1.43(1.28-1.60)**	**0.75(0.59-0.97)**	**2.22(1.84-2.68)**	**2.30(1.40-3.76)**
≥80	1.10(0.99-1.23)	**6.08(5.55-6.67)**	**0.41(0.29-0.59)**	**5.85(4.92-6.96)**	**4.48(2.37-8.47)**
**Marriage**					
In marriage	1.00 [Ref.]	1.00 [Ref.]	1.00 [Ref.]	1.00 [Ref.]	1.00 [Ref.]
Not in marriage	**0.85(0.77-0.94)**	**1.64(1.54-1.74)**	0.98(0.75-1.29)	**1.25(1.09-1.43)**	1.10(0.66-1.85)
**Residence**					
Rural	1.00 [Ref.]	1.00 [Ref.]	1.00 [Ref.]	1.00 [Ref.]	1.00 [Ref.]
Town	**1.21(1.10-1.33)**	0.99(0.94-1.05)	1.06(0.80-1.40)	1.00(0.86-1.16)	0.93(0.54-1.59)
City	**1.36(1.21-1.53)**	**1.12(1.05-1.20)**	**1.39(1.02-1.89)**	0.94(0.77-1.14)	**0.43(0.21-0.88)**
**SES**					
Low SES	1.00 [Ref.]	1.00 [Ref.]	1.00 [Ref.]	1.00 [Ref.]	1.00 [Ref.]
Medium SES	**1.14(1.03-1.26)**	**0.94(0.89-0.99)**	0.76(0.56-1.03)	1.03(0.89-1.20)	1.18(0.65-2.15)
High SES	**1.15(1.05-1.26)**	**0.86(0.81-0.90)**	1.07(0.84-1.36)	0.87(0.76-1.01)	1.22(0.75-1.98)
**Regular physical activity**					
Yes	1.00 [Ref.]	1.00 [Ref.]	1.00 [Ref.]	1.00 [Ref.]	1.00 [Ref.]
No	**1.11(1.02-1.21)**	**1.19(1.12-1.25)**	1.10(0.87-1.38)	1.13(0.98-1.29)	0.78(0.49-1.25)
**Smoking**					
Non-smoker	1.00 [Ref.]	1.00 [Ref.]	1.00 [Ref.]	1.00 [Ref.]	1.00 [Ref.]
Smoker	**1.18(1.07-1.31)**	1.03(0.96-1.09)	1.05(0.79-1.41)	1.02(0.87-1.19)	**2.23(1.20-4.13)**

**Alcohol drinking**					
Non-drinker	1.00 [Ref.]	1.00 [Ref.]	1.00 [Ref.]	1.00 [Ref.]	1.00 [Ref.]
Drinker	1.00(0.90-1.10)	1.01(0.95-1.07)	0.86(0.65-1.15)	1.06(0.92-1.24)	1.12(0.62-2.01)
**Sleep duration**					
≤5.0h	**1.23(1.08-1.41)**	0.97(0.90-1.05)	0.89(0.61-1.31)	1.14(0.93-1.39)	0.87(0.39-1.96)
5.1-7.0h	**1.21(1.09-1.33)**	0.94(0.88-1.00)	1.01(0.78-1.32)	0.97(0.82-1.13)	1.24(0.74-2.09)
7.1-8.0h	1.00 [Ref.]	1.00 [Ref.]	1.00 [Ref.]	1.00 [Ref.]	1.00 [Ref.]
>8.0h	**1.12(1.01-1.24)**	**1.14(1.08-1.20)**	0.90(0.67-1.21)	**1.21(1.04-1.41)**	1.15(0.66-2.03)
**BMI categories**					
Underweight	**0.88(0.80-0.96)**	**1.19(1.14-1.25)**	0.75(0.54-1.02)	**1.21(1.06-1.38)**	1.21(0.67-2.19)
Normal	1.00 [Ref.]	1.00 [Ref.]	1.00 [Ref.]	1.00 [Ref.]	1.00 [Ref.]
Overweight	**1.44(1.28-1.62)**	**0.81(0.74-0.89)**	**1.42(1.07-1.88)**	0.94(0.77-1.16)	1.22(0.69-2.14)
Obesity	**1.30(1.02-1.64)**	1.10(0.92-1.32)	**1.76(1.03-2.99)**	**0.52(0.31-0.89)**	1.16(0.35-3.85)

Abbreviations: SES, socioeconomic status; BMI, body mass index (calculated as weight in kilograms divided by square of height in meters); HR, hazard [illegible]

STable 18. Role of factors in transitions between cardiometabolic conditions and mortality using age as the time scale (n=13933)

Factors	HR (95% CI)				
	**A(healthy→first disease)**	**B(healthy→mortality)**	**C(first disease→ multimorbidity)**	**D(first disease→ mortality)**	**E(multimorbidity→ mortality)**
**Gender**					
Male	1.00 [Ref.]	1.00 [Ref.]	1.00 [Ref.]	1.00 [Ref.]	1.00 [Ref.]
Female	**1.20(1.09-1.31)**	**0.80(0.75-0.84)**	1.27(0.99-1.63)	0.87(0.75-1.01)	1.24(0.73-2.10)
**Marriage**					
In marriage	1.00 [Ref.]	1.00 [Ref.]	1.00 [Ref.]	1.00 [Ref.]	1.00 [Ref.]
Not in marriage	**0.35(0.32-0.38)**	**1.59(1.49-1.69)**	**0.41(0.32-0.53)**	**1.25(1.10-1.43)**	1.16(0.72-1.86)
**Residence**					
Rural	1.00 [Ref.]	1.00 [Ref.]	1.00 [Ref.]	1.00 [Ref.]	1.00 [Ref.]
Town	1.08(0.98-1.19)	1.00(0.95-1.06)	0.98(0.75-1.30)	1.08(0.93-1.25)	0.94(0.56-1.58)
City	1.07(0.95-1.19)	1.02(0.96-1.09)	1.22(0.90-1.64)	0.94(0.77-1.14)	**0.47(0.23-0.96)**
**SES**					
Low SES	1.00 [Ref.]	1.00 [Ref.]	1.00 [Ref.]	1.00 [Ref.]	1.00 [Ref.]
Medium SES	0.98(0.89-1.08)	0.97(0.92-1.02)	**0.66(0.49-0.89)**	1.11(0.95-1.28)	1.34(0.75-2.37)
High SES	**1.09(1.00-1.19)**	**0.92(0.87-0.97)**	0.95(0.76-1.19)	0.97(0.84-1.12)	1.24(0.76-2.02)
**Regular physical activity**					
Yes	1.00 [Ref.]	1.00 [Ref.]	1.00 [Ref.]	1.00 [Ref.]	1.00 [Ref.]
No	0.92(0.85-1.00)	**1.22(1.16-1.28)**	0.88(0.70-1.09)	**1.25(1.09-1.42)**	0.85(0.54-1.36)
**Smoking**					
Non-smoker	1.00 [Ref.]	1.00 [Ref.]	1.00 [Ref.]	1.00 [Ref.]	1.00 [Ref.]
Smoker	**1.19(1.07-1.31)**	**1.08(1.02-1.15)**	0.94(0.71-1.24)	1.12(0.96-1.31)	**2.63(1.45-4.75)**
**Alcohol drinking**					
Non-drinker	1.00 [Ref.]	1.00 [Ref.]	1.00 [Ref.]	1.00 [Ref.]	1.00 [Ref.]
Drinker	0.92(0.83-1.01)	1.02(0.96-1.08)	0.78(0.59-1.04)	1.12(0.96-1.30)	1.02(0.57-1.83)
**Sleep duration**					
≤5h	1.02(0.90-1.16)	1.05(0.97-1.13)	0.75(0.52-1.09)	**1.28(1.05-1.57)**	0.91(0.41-2.02)
>5-7h	1.07(0.97-1.17)	0.98(0.92-1.04)	0.92(0.72-1.19)	1.06(0.90-1.24)	1.37(0.83-2.27)
>7-8h	1.00 [Ref.]	1.00 [Ref.]	1.00 [Ref.]	1.00 [Ref.]	1.00 [Ref.]
>8h	**0.80(0.73-0.88)**	**1.13(1.06-1.19)**	**0.63(0.47-0.84)**	**1.30(1.12-1.51)**	1.25(0.72-2.18)

**BMI**					
Underweight	**0.67(0.61-0.73)**	**1.15(1.10-1.21)**	**0.58(0.42-0.79)**	**1.21(1.06-1.39)**	1.07(0.59-1.94)
Normal	1.00 [Ref.]	1.00 [Ref.]	1.00 [Ref.]	1.00 [Ref.]	1.00 [Ref.]
Overweight	**1.49(1.33-1.67)**	**0.86(0.78-0.94)**	**1.67(1.27-2.20)**	0.93(0.75-1.14)	1.06(0.62-1.80)
Obesity	**1.42(1.13-1.79)**	1.05(0.88-1.25)	**2.04(1.20-3.47)**	**0.53(0.31-0.90)**	1.05(0.32-3.46)

Abbreviations: SES, socioeconomic status; BMI, body mass index (calculated as weight in kilograms divided by square of height in meters); HR, hazard ratio.

STable 19. Role of factors in transitions between cardiometabolic conditions and mortality using three smoking status (n=13933)

Factors	HR (95% CI)				
	**A(healthy→first disease)**	**B(healthy→ mortality)**	**C(first disease→ multimorbidity)**	**D(first disease→ mortality)**	**E(multimorbidity→ mortality)**
**Gender**					
Male	1.00 [Ref.]	1.00 [Ref.]	1.00 [Ref.]	1.00 [Ref.]	1.00 [Ref.]
Female	**1.24(1.13-1.36)**	**0.84(0.80-0.89)**	**1.39(1.07-1.81)**	**0.84(0.73-0.98)**	1.24(0.73-2.13)
**Age (y)**					
60-69	1.00 [Ref.]	1.00 [Ref.]	1.00 [Ref.]	1.00 [Ref.]	1.00 [Ref.]
70-79	**1.28(1.16-1.41)**	**1.42(1.28-1.59)**	**0.75(0.58-0.97)**	**2.20(1.83-2.65)**	**2.29(1.40-3.75)**
≥80	1.07(0.96-1.19)	**6.01(5.48-6.59)**	**0.41(0.28-0.58)**	**5.81(4.89-6.91)**	**4.50(2.37-8.54)**
**Marriage**					
In marriage	1.00 [Ref.]	1.00 [Ref.]	1.00 [Ref.]	1.00 [Ref.]	1.00 [Ref.]
Not in marriage	**0.85(0.77-0.94)**	**1.64(1.54-1.74)**	0.97(0.74-1.28)	**1.25(1.09-1.44)**	1.10(0.66-1.86)
**Residence**					
Rural	1.00 [Ref.]	1.00 [Ref.]	1.00 [Ref.]	1.00 [Ref.]	1.00 [Ref.]
Town	**1.19(1.08-1.31)**	0.99(0.93-1.05)	1.06(0.80-1.40)	0.99(0.85-1.16)	0.91(0.53-1.57)
City	**1.32(1.17-1.48)**	**1.11(1.04-1.19)**	**1.40(1.02-1.90)**	0.93(0.77-1.13)	**0.44(0.22-0.88)**
**SES**					
Low SES	1.00 [Ref.]	1.00 [Ref.]	1.00 [Ref.]	1.00 [Ref.]	1.00 [Ref.]
Medium SES	**1.14(1.03-1.25)**	**0.94(0.89-0.99)**	0.75(0.55-1.02)	1.03(0.89-1.19)	1.19(0.65-2.17)
High SES	**1.15(1.06-1.26)**	**0.85(0.81-0.90)**	1.06(0.84-1.35)	0.87(0.75-1.00)	1.24(0.76-2.03)
**Regular physical activity**					
Yes	1.00 [Ref.]	1.00 [Ref.]	1.00 [Ref.]	1.00 [Ref.]	1.00 [Ref.]
No	**1.11(1.02-1.21)**	**1.19(1.13-1.25)**	1.11(0.88-1.40)	1.12(0.98-1.28)	0.78(0.49-1.25)
**Smoking**					
Non-smoker	1.00 [Ref.]	1.00 [Ref.]	1.00 [Ref.]	1.00 [Ref.]	1.00 [Ref.]
Former smoker	**1.26(1.09-1.45)**	**1.19(1.10-1.29)**	1.25(0.85-1.85)	0.98(0.80-1.20)	1.99(0.93-4.24)
Current smoker	**1.15(1.03-1.28)**	0.94(0.88-1.01)	0.96(0.70-1.33)	1.03(0.87-1.23)	**2.37(1.21-4.63)**
**Alcohol drinking**					
Non-drinker	1.00 [Ref.]	1.00 [Ref.]	1.00 [Ref.]	1.00 [Ref.]	1.00 [Ref.]
Drinker	1.00(0.91-1.11)	1.00(0.95-1.07)	0.86(0.64-1.15)	1.06(0.91-1.23)	1.10(0.61-1.98)
**Sleep duration**					
≤5.0h	**1.21(1.06-1.38)**	0.97(0.89-1.05)	0.88(0.60-1.29)	1.13(0.92-1.39)	0.85(0.38-1.92)

5.1-7.0h	**1.19(1.08-1.31)**	0.94(0.88-1.00)	1.00(0.77-1.31)	0.96(0.82-1.13)	1.21(0.72-2.04)
7.1-8.0h	1.00 [Ref.]	1.00 [Ref.]	1.00 [Ref.]	1.00 [Ref.]	1.00 [Ref.]
>8.0h	**1.10(1.00-1.22)**	**1.13(1.07-1.20)**	0.90(0.67-1.21)	**1.21(1.04-1.40)**	1.13(0.64-2.00)
**BMI categories**					
Underweight	**0.86(0.79-0.95)**	**1.19(1.14-1.25)**	0.74(0.54-1.02)	**1.20(1.06-1.37)**	1.20(0.66-2.17)
Normal	1.00 [Ref.]	1.00 [Ref.]	1.00 [Ref.]	1.00 [Ref.]	1.00 [Ref.]
Overweight	**1.47(1.31-1.64)**	**0.81(0.73-0.89)**	**1.41(1.07-1.87)**	0.94(0.76-1.16)	1.22(0.69-2.15)
Obesity	**1.33(1.05-1.67)**	1.10(0.92-1.32)	**1.79(1.05-3.05)**	**0.52(0.31-0.88)**	1.14(0.34-3.80)

Abbreviations: SES, socioeconomic status; BMI, body mass index (calculated as weight in kilograms divided by square of height in meters); HR, hazard
